# Prevalence and prescribing patterns of oral corticosteroids in the United States, Taiwan, and Denmark, 2009–2018

**DOI:** 10.1111/cts.13649

**Published:** 2023-10-06

**Authors:** Beth I. Wallace, Hui‐Ju Tsai, Paul Lin, Kristian Aasbjerg, Ann Chen Wu, Yi‐Fen Tsai, Christian Torp‐Pedersen, Akbar K. Waljee, Tsung‐Chieh Yao

**Affiliations:** ^1^ University of Michigan Ann Arbor Michigan USA; ^2^ Center for Clinical Management Research Lieutenant Colonel Charles S. Kettles VA Medical Center Ann Arbor Michigan USA; ^3^ Institute for Healthcare Policy and Innovation Ann Arbor Michigan USA; ^4^ Institute of Population Health Sciences National Health Research Institutes Zhunan Taiwan; ^5^ National Tsing‐Hua University College of Life Science Hsinchu Taiwan; ^6^ Himmerland Eye Clinic Aalborg Denmark; ^7^ Harvard Medical School Boston Massachusetts USA; ^8^ Department of Pediatrics, Children's Hospital Boston Massachusetts USA; ^9^ Department of Clinical Investigation and Cardiology, Nordsjaellands Hospital Hilleroed Denmark; ^10^ Department of Clinical Medicine, Faculty of Health and Medical Sciences University of Copenhagen Copenhagen Denmark; ^11^ Division of Allergy, Asthma, and Rheumatology, Department of Pediatrics, Chang Gung Memorial Hospital Taoyuan Taiwan; ^12^ School of Medicine Chang Gung University College of Medicine Taoyuan Taiwan

## Abstract

Oral corticosteroids (OCS) are commonly prescribed for acute, self‐limited conditions, despite studies demonstrating toxicity. Studies evaluating longitudinal OCS prescribing in the general population are scarce and do not compare use across countries. This study investigated and compared OCS prescription patterns from 2009 to 2018 in the general populations of the United States, Taiwan, and Denmark. This international population‐based longitudinal cohort study used nationwide claims databases (United States: Optum Clinformatics Data Mart; de‐identified; Taiwan: National Health Insurance Research Database; and Denmark: National Prescription and Patient Registries/Danish National Patient Registry) to evaluate OCS prescribing. We classified annual OCS duration as short‐term (1–29 days), medium‐term (30–89 days), or long‐term (≥90 days). Longitudinal change in annual prevalence of OCS use and physician prescribing patterns were reported. Among 54,630,437 participants, average annual percentage of overall OCS use was 6.8% in the United States, 17.5% in Taiwan, and 2.2% in Denmark during 2009–2018. Prevalence of OCS prescribing increased at an average annual rate of 0.1%–0.17%, mainly driven by short‐term prescribing to healthy adults. One‐quarter to one‐fifth of OCS prescribing was associated with a diagnosis of respiratory infection. Family practice and internal medicine physicians were among the highest OCS prescribers across countries and durations. Age‐ and sex‐stratified trends mirrored unstratified trends. This study provides real‐world evidence of an ongoing steady increase in OCS use in the general populations of the United States, Taiwan, and Denmark. This increase is largely driven by short‐term OCS prescribing to healthy adults, a practice previously viewed as safe but recently shown to incur substantial population‐level risk.

**Study Highlights**

**WHAT IS THE CURRENT KNOWLEDGE ON THIS TOPIC?**

Oral corticosteroids (OCS) are commonly prescribed for acute, self‐limited conditions. Studies evaluating longitudinal OCS prescribing in the general population are scarce and do not compare use across countries.

**WHAT QUESTION DID THIS STUDY ADDRESS?**

This population‐based longitudinal cohort study used claims databases from the United States, Taiwan, and Denmark to evaluate current practice patterns and recent trends in OCS prescribing. The analysis included 54,630,437 enrollees during the period 2009–2018.

**WHAT DOES THIS STUDY ADD TO OUR KNOWLEDGE?**

Average annual percentage of OCS use was 6.8% in the United States, 17.5% in Taiwan, and 2.2% in Denmark, increasing 0.1–0.17% annually. Most prescriptions were short bursts (1–29 days) prescribed to healthy adults for self‐limited conditions, like respiratory infections.

**HOW MIGHT THIS CHANGE CLINICAL PHARMACOLOGY OR TRANSLATIONAL SCIENCE?**

This study demonstrates an ongoing steady increase in OCS prescribing in the general populations of the United States, Taiwan, and Denmark. These trends were driven by short‐term OCS prescribing to healthy adults for self‐limited conditions, a practice recently shown to incur substantial population‐level risk.

## INTRODUCTION

Since their discovery 70 years ago, oral corticosteroids (OCS) have been widely used to treat chronic conditions, including cancers, autoimmune diseases, obstructive pulmonary diseases, and organ transplantation.^1^ Such use remains common today, despite mounting evidence of dose‐dependent toxicity associated with prolonged OCS use.^2^, ^3^, ^4^, ^5^ Although short‐term OCS use (<30 days)^6^ has historically been of less concern, recent nationwide studies in the United States and Taiwan have demonstrated associations between short‐term OCS and an increased risk of serious adverse events, including sepsis, pneumonia, gastrointestinal bleeding, venous thromboembolism, heart failure, and fracture.^6^, ^7^, ^8^ In the same studies, one‐fifth to one‐quarter of individuals in the general populations of both countries were prescribed OCS over a 3‐year period. Furthermore, one‐third to half of OCS prescriptions in these studies were associated with acute, self‐limited conditions, such as upper respiratory tract infections, a finding supported by prior work in the general population of the United States.^6^, ^9^, ^10^, ^11^, ^12^ These striking data demonstrate that short‐term OCS use is both common among otherwise healthy patients, and associated with rare but severe adverse events.

Several previous studies address the prevalence of OCS use in the general population. However, these focus on long‐term OCS use (≥90 days), and most do not include estimates from the past decade. For example, cross‐sectional studies prior to 2008 in the United Kingdom and Iceland estimated that 0.5%–0.9% of those populations used long‐term OCS,^13^ with a median duration of treatment ranging from 7 months to 3 years.^14^, ^15^, ^16^, ^17^ Data from the National Health and Nutrition Examination Surveys from 1999–2008 found an overall prevalence of OCS use of 1.2% among adults in the United States, with approximately two‐thirds of users receiving long‐term OCS.^18^ However, a more recent publication by Benard‐Laribière et al. reported increasing prevalence of overall OCS use from 14.7 in 2007 to 17.1% in 2014 in a longitudinal cohort of French adults.^19^ This study, along with the national findings in United States and Taiwan highlighted above, support the hypothesis that global prescribing of OCS may have increased in the general population over the last decade.

To our knowledge, studies evaluating longitudinal trends and prescription patterns of OCS in the general population are scarce, do not compare prevalence of OCS use across countries, and under‐represent Asian countries. Utilizing data derived from three nationwide cohorts, this study aimed to investigate and compare longitudinal trends and prescription patterns of OCS from 2009 to 2018 in the general populations of three countries: the United States, Taiwan, and Denmark.

## METHODS

### Study design and data sources

This international population‐based longitudinal cohort study used nationwide claims databases from the United States (Optum Clinformatics Data Mart; de‐identified), Taiwan (National Health Insurance Research Database),^7^, ^18^ and Denmark (National Prescription and Patient Registries/Danish National Patient Registry)^19^, ^20^ to evaluate OCS prescribing. All information used in this study was derived from de‐identified medical and pharmacy claims data in United States, Taiwan, and Denmark. Briefly, Optum in the United States is a commercial insurance program containing data for ~87 million individuals between 2001 and 2020, with 15–20 million active members annually. The National Health Insurance Research Database in Taiwan is a single‐payer mandatory enrollment insurance program containing data for ~23 million individuals (more than 99% of the Taiwanese population) between 2009 and 2018. The National Prescription and Patient Registries/Danish National Patient Registry in Denmark is a nationwide hospital registry system containing data for ~5.5 million individuals between 2009 and 2018. This study was approved or deemed exempt by the Institutional Review Boards of the University of Michigan (United States), the National Health Research Institutes (Taiwan), and the Danish Data Protection Agency (Denmark).

### Study participants

For each year of the study period, we classified all enrolled individual with greater than or equal to one pharmacy claim as the total number of subjects enrolled in that year (our denominator). OCS users in that given year included all patients enrolled during that year who had greater than or equal to one pharmacy claim for OCS (our numerator). Non‐users in that given year had greater than or equal to one pharmacy claim during this same period, but no claims for systemic (oral or injectable) corticosteroids. We defined an OCS user's index date as the date of their first pharmacy claim for OCS use during the study period, and a non‐user's index date as the first pharmacy claim of any kind made except OCS during the study period. Prevalence of OCS users for each year of the study period was calculated as follows: (OCS users in that year)/(total number of subjects enrolled in that year).

We limited our analysis to individuals with greater than or equal to 6 months of medical and pharmacy claims (for OCS users) or greater than or equal to 6 months of medical claims (for non‐users) prior to index date to ensure adequate capture of baseline comorbid conditions. We captured these conditions using the Elixhauser Comorbidity Index, a composite comorbidity score of individuals based on International Classification of Disease (ICD) diagnosis codes and healthcare utilization.^20^, ^21^ We excluded individuals with claims for injectable but not OCS, to avoid issues with misclassification of injectable corticosteroids in claims data. We also excluded individuals who died during the study period to reduce immortal time bias. Cohort selection criteria are outlined in Figure S1.

### Oral corticosteroid utilization

To assess OCS utilization, we standardized all oral corticosteroid dosages to prednisone equivalents, and used pharmacy claims data to derive average daily dose, cumulative annual dose, and cumulative annual duration (Table S1).^22^ Dose conversion to oral prednisone equivalents was performed per standard practice, to account for variation in potency and international utilization across different OCS formulations.^6^, ^7^ We classified annual duration of OCS into three categories for each year: short‐ (1–29 days' supply), medium‐ (30–89 days' supply), and long‐term (≥90 days' supply).^6^, ^13^

### Indication and physician specialty

To evaluate medical diagnoses associated with OCS use, we linked each pharmacy claim for OCS to primary medical claims occurring in the 30 days prior to the OCS dispense date. In the United States and Taiwan, we captured diagnoses made prior to October 1, 2015 (United States) or December 31, 2015 (Taiwan) using ICD, ninth revision (ICD‐9) codes, and those made thereafter using tenth revision (ICD‐10) codes. ICD‐10 codes were used throughout the period 2009–2018 in Denmark. We grouped ICD codes into clinically meaningful categories with Agency for Healthcare Research and Quality Clinical Classification Software (CCS), using CCS version 2015 for ICD‐9, and CCS Refined (CCSR) version 2022.21 for ICD‐10.^1^ We used Medical Expenditure Panel Survey (MEPS) categories to compare data across CCS and CCSR groupings.^23^ To assess physician specialties in the United States and Taiwan, we used data linked to pharmacy claims to identify the specialty of each physician responsible for an OCS prescription during the study period.

### Statistical analysis

Baseline demographic and clinical characteristics were outlined using descriptive statistics. Of note, OCS dose and duration data were not available in Denmark. We calculated the annual prevalence of overall, short‐, medium‐, and long‐term OCS use among individuals with any claim during 2009–2018. We also estimated the annual prevalence of OCS use stratified by age and sex separately. We used linear regression to evaluate time trends in prevalence of OCS use annually during this period. A *p* value less than 0.05 was declared statistically significant. All analyses were conducted with SAS version 9.4 (SAS Institute).

## RESULTS

Table 1 indicates the baseline characteristics of the populations in the United States, Taiwan, and Denmark. A total of 54,630,437 participants (28,386,692, 52.0% women) were included. In all countries, OCS users were older and more often women than nonusers. OCS users in Taiwan had higher comorbidity scores than those in the United States (mean [SD] Elixhauser Comorbidity Index: 1.1 [1.7] in the United States vs. 2.1 [2.4] in Taiwan). A substantial proportion of OCS users had Elixhauser Comorbidity Index equal to 0 in both the United States and Taiwan (53.8% in the United States and 31.9% in Taiwan). OCS users had higher outpatient healthcare utilization than nonusers in all countries; median (interquartile range [IQR]) outpatient visits for OCS users versus non‐users were 4 (IQR: 1–7) versus 1 (IQR: 0–3) in the United States, 5 (IQR: 2–11) versus 0 (0–3) in Taiwan, and 1 (IQR: 0–4) versus 0 (IQR: 0–1) in Denmark, respectively. The median per individual user annual number of OCS prescriptions was similar across countries (median [IQR]: 1.0 [1.0–2.0] in the United States; 1.3 [1.0–2.0] in Taiwan; and 1.0 [1.0–2.0] in Denmark).

**TABLE 1:** Baseline demographic and clinical characteristics of study participants in the United States, Taiwan, and Denmark.

Characteristic	USA		Taiwan		Denmark	
	OCS users	Nonusers	OCS users	Nonusers	OCS users	Nonusers
	*N =* 7,369,654	*N* = 19,306,222	*N* = 14,103,070	*N* = 8,359,873	*N* = 460,775	*N* = 5,030,843
Age, mean (SD), years	43.7 (21.6)	37.7 (21.5)	36.4 (20.7)	29.8 (20.7)	49.3 (18.6)	32.0 (22.1)
Female, *n* (%)	4,092,723 (55.5%)	10,182,870 (52.7%)	7,395,492 (52.4)	3,925,700 (47.0)	261,734 (56.8)	2,528,173 (50.3)
Elixhauser Comorbidity Index score 6 month prior to index date, *n* (%)						
Mean (SD)	1.1 (1.7)	0.6 (1.3)	2.1 (2.4)	0.7 (1.6)	– (–)	– (–)
0	3,964,481 (53.8)	13,130,433 (68.0)	4,494,458 (31.9)	6,082,772 (72.8)	– (–)	– (–)
1	1,520,396 (20.6)	3,194,915 (16.5)	3,139,000 (22.3)	852,145 (10.2)	– (–)	– (–)
2	808,756 (11.0)	1,509,523 (7.8)	1,999,316 (14.2)	494,548 (5.9)	– (–)	– (–)
3+	1,076,021 (14.6)	1,471,351 (7.6)	4,470,296 (31.7)	930,408 (11.1)	– (–)	– (–)
Healthcare utilization up to one year prior to index date, median (IQR)						
Median (IQR) outpatient visits	4.0 (1.0–7.0)	1.0 (0.0–3.0)	5 (2–11)	0.0 (0.0–3.0)	1 (0–4)	0 (0–1)
Median (IQR) hospitalizations	0.0 (0.0–0.0)	0.0 (0.0–0.0)	0 (0–0)	0.0 (0.0–0.0)	0 (0–2)	0 (0–0)
Oral corticosteroid prescriptions, annual^a^						
Median (IQR) number of prescriptions per year	1.0 (1.0–2.0)	– (–)	1.3 (1.0–2.0)	– (–)	1.0 (1.0–2.0)	– (–)
Median (IQR) dose per prescription, mg^b^	140.0 (105.0–240.0)	– (–)	40.0 (30.0–52.5)	– (–)	– (–)	– (–)
Median (IQR) cumulative dose, mg	200.0 (105.0–310.0)	– (–)	58.2 (37.5–97.5)	– (–)	– (–)	– (–)
Median (IQR) cumulative duration, days	6.0 (5.0–12.0)	– (–)	5.0 (3.0–9.0)	– (–)	– (–)	– (–)

Abbreviation: IQR, interquartile range; OCS, oral corticosteroid; SD, standard deviation.

^a^ Among patients who were prescribed corticosteroids in a given year.

^b^ Reported in prednisone equivalents.

**FIGURE 1:**
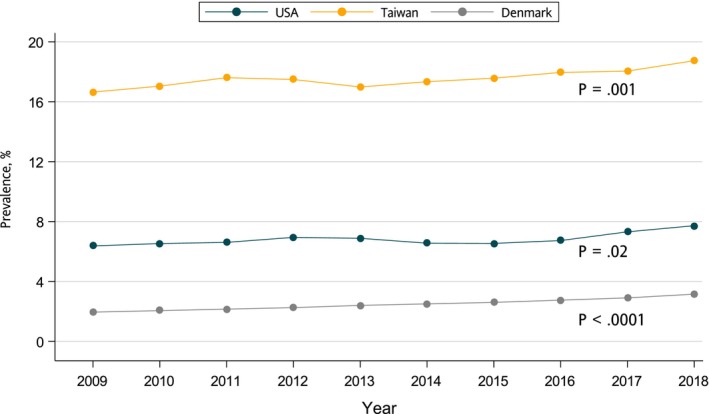
Ten‐year trend on prevalence of overall oral corticosteroid use in USA, Taiwan, and Denmark.

**FIGURE 2:**
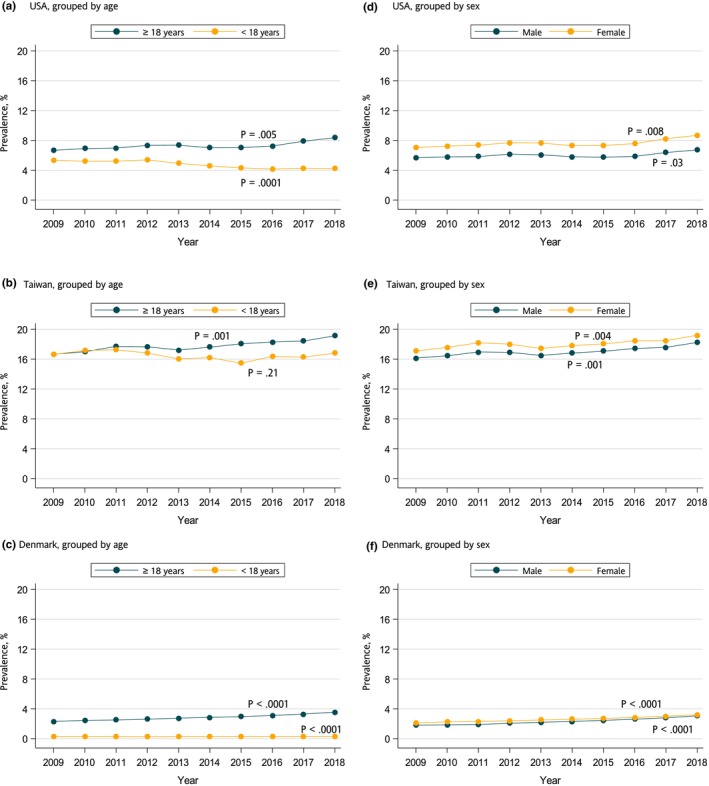
Ten‐year trend on prevalence of overall oral corticosteroid use in the United States, Taiwan, and Denmark, stratified by age (a–c) and stratified by sex (d–f).

Figure 1 presents the prevalence and time trends in OCS use over the 10‐year study period. The average annual prevalence of overall OCS use was 6.8% in the United States, 17.5% in Taiwan, and 2.2% in Denmark. The overall prevalence of OCS use significantly increased during the study period in all three countries (in the United States: from 6.4% to 7.7%, *β* = 0.1, *p*_for linear trend_ = 0.02; in Taiwan: from 16.6% to 18.7%, *β* = 0.17, *p*_for linear trend_ = 10^−3^; and in Denmark: from 1.7% to 2.9%, *β* = 0.13, *p*_for linear trend_ < 10^−3^). When stratified by age and sex, increasing trends were observed across adults of both sexes, but not in children under age 18 (Figure 2). Increasing OCS prevalence over the study period was primarily driven by short‐term prescriptions in the United States, whereas short‐, medium‐, and long‐term prescriptions all increased in Taiwan (Figure S2). Trends similar to those shown for overall use were observed for short‐, medium‐, and long‐term use when stratified by age and sex (Figures S3 and S4).

For overall OCS use, the median (IQR) of both annual dose and duration were higher in the United States (200.0 [105.0–310.0] mg and 6.0 [5.0–12.0] days) than in Taiwan (58.2 [37.5–97.5] mg and 5.0 [3.0–9.0] days; Table 1). The exact dose and duration in Denmark are not available. Median dose and duration for short‐, medium‐, and long‐term prescription remained higher in the United States than those in Taiwan, except for the duration for medium‐term prescriptions (Table S2). Most OCS prescriptions were for prednisone or prednisolone (63.9% in the United States; 54.4% in Taiwan; and 78.4% in Denmark). Methylprednisolone accounted for 31.3% of OCS prescriptions in the United States, 12.5% in Taiwan, and 7.7% in Denmark. Dexamethasone accounted for 3.7% of OCS prescriptions in the United States, 23.1% in Taiwan, and less than or equal to 1% in Denmark (Table S3).

Table 2 shows the top 10 indications for overall OCS use in each country during the study period. Acute bronchitis and upper respiratory infection were the most common indications in the United States and Taiwan, accounting for 18.7% and 25.1% of all OCS prescriptions, respectively. Chronic obstructive pulmonary disease (COPD), asthma, and other respiratory conditions were the most common indications in Denmark, accounting for 17.0% of all prescriptions. Five common indications among the top 10 were observed across all countries: COPD, asthma, and other respiratory conditions; allergic reactions; osteoarthritis and other non‐traumatic joint disorders; systemic lupus and connective tissue disorders; and skin disorders. In the United States and Taiwan, the top 10 indications for short‐ and medium‐term of OCS use were comparable to those for overall OCS use. Osteoarthritis and non‐traumatic joint disorders, COPD, asthma, and other respiratory conditions, and systemic lupus and connective tissue disorders were the top three indications for long‐term OCS use in both the United States and Taiwan, cumulatively accounting for 34.7% (the United States) and 45.7% (Taiwan) of long‐term prescriptions during the study period. The top 10 indications for OCS use in each year of the study period are summarized in Tables [Link], [Link]. In the United States, overall and short‐term OCS use for COPD and asthma and allergic reactions declined over the study period, whereas use for skin and joint disorders increased. Similar trends were seen in Taiwan, with exception of an increase in prescribing for acute rather than chronic respiratory conditions. In contrast, long‐term prescribing for both joint disorders and chronic lung conditions declined over the study period in both countries, whereas prescribing for acute lung conditions and allergic reactions remained stable. Long‐term OCS prescribing for connective tissue disorders declined in both countries across all durations.

**TABLE 2:** Top 10 indications of overall, short‐, medium‐ and long‐term oral corticosteroid use in the United States, Taiwan, and Denmark.

USA^a^		Taiwan^a^		Denmark^a^	
Top 10 indications	%	Top 10 indications	%	Top 10 indications	%
Overall use					
Acute bronchitis and URI	18.7	Acute bronchitis and URI	25.1	COPD, asthma, and other respiratory conditions	17.0
COPD, asthma, and other respiratory conditions	16.3	Allergic reactions	19.6	Nervous system disorders	11.9
Back problems	7.3	COPD, asthma, and other respiratory conditions	12.4	Osteoarthritis and other non‐traumatic joint disorders	10.2
Allergic reactions	6.0	Skin disorders	10.4	Allergic reactions	6.7
Osteoarthritis and other non‐traumatic joint disorders	5.9	Osteoarthritis and other non‐traumatic joint disorders	5.2	Pneumonia	6.3
Skin disorders	3.7	Systemic lupus and connective tissue disorders	3.4	Systemic lupus and connective tissue disorders	6.3
Systemic lupus and connective tissue disorders	3.2	Tonsillitis	2.5	Skin disorders	4.2
Nervous system disorders	2.8	Trauma‐related disorders	1.7	Cancer	4.1
Trauma‐related disorders	2.0	Infectious diseases	1.6	Other endocrine, nutritional, and immune disorders	4.0
Hypertension	1.9	Back problems	1.2	Heart disease	3.5
Short‐term use					
Acute bronchitis and URI	21.3	Acute bronchitis and URI	30.5		
COPD, asthma, and other respiratory conditions	16.5	Allergic reactions	22.8		
Back problems	8.1	Skin disorders	11.5		
Allergic reactions	6.6	COPD, asthma, and other respiratory conditions	10.6		
Osteoarthritis and other non‐traumatic joint disorders	4.7	Tonsillitis	3.2		
Skin disorders	3.8	Trauma‐related disorders	3.0		
Nervous system disorders	2.8	Infectious diseases	2.6		
Systemic lupus and connective tissue disorders	2.6	Osteoarthritis and other non‐traumatic joint disorders	2.2		
Trauma‐related disorders	2.3	Nervous system disorders	1.9		
Otitis media and related conditions	2.1	Systemic lupus and connective tissue disorders	1.3		
Medium‐term use					
COPD, asthma, and other respiratory conditions	20.3	Acute bronchitis and URI	20.2		
Acute bronchitis and URI	10.5	COPD, asthma, and other respiratory conditions	18.6		
Osteoarthritis and other non‐traumatic joint disorders	8.0	Allergic reactions	18.0		
Back problems	5.0	Skin disorders	11.6		
Allergic reactions	5.0	Osteoarthritis and other non‐traumatic joint disorders	7.8		
Cancer	4.4	Other endocrine, nutritional, and immune disorders	3.2		
Skin disorders	4.1	Systemic lupus and connective tissue disorders	2.0		
Systemic lupus and connective tissue disorders	3.2	Back problems	1.8		
Other stomach and intestinal disorders	3.0	Tonsillitis	1.5		
Other endocrine, nutritional, and immune disorder	2.8	Trauma‐related disorders	1.4		
Long‐term use					
Osteoarthritis and other non‐traumatic joint disorders	14.8	Osteoarthritis and other non‐traumatic joint disorders	18.5		
COPD, asthma, and other respiratory conditions	10.3	Systemic lupus and connective tissue disorders	13.8		
Systemic lupus and connective tissue disorders	9.6	COPD, asthma, and other respiratory conditions	13.4		
Cancer	4.3	Allergic reactions	6.5		
Other endocrine, nutritional, and immune disorders	3.4	Kidney diseases	5.4		
Skin disorders	3.3	Other endocrine, nutritional, and immune disorder	5.3		
Other stomach and intestinal disorders	3.1	Acute bronchitis and URI	5.2		
Hypertension	2.6	Skin disorders	5.2		
Nervous system disorders	2.6	Cancer	2.8		
Acute bronchitis and URI	2.5	Hypertension	2.3		

Abbreviations: COPD, chronic obstructive pulmonary disease; URI, upper respiratory infection.

^a^ Denominator includes all oral corticosteroid prescriptions dispensed during the study period, regardless of prescribing provider type.

Table 3 outlines the top five physician specialties responsible for OCS prescribing in the United States and Taiwan during the study period. In the United States, these were family practice (31.6%), internal medicine (15.8%), emergency medicine (8.2%), pediatrics (6.7%), and rheumatology (4.6%). In Taiwan, these were dermatology (22.0%), family practice (19.3%), otolaryngology (14.8%), internal medicine (11.8%), and pediatrics (11.2%). After stratifying by duration of OCS use, family practice and internal medicine remained among the top five specialties across all durations in both countries. In the United States, emergency medicine, pediatrics, and surgery were among the five specialties most likely to prescribe short‐term OCS use. Rheumatology and pulmonology were among the top five specialties for both medium‐ and long‐term use; rheumatologists prescribed 8.6% of medium‐term and 31.6% of long‐term prescriptions, whereas pulmonologists prescribed 5.8% of medium‐term and 5.8% of long‐term prescriptions. In Taiwan, dermatology was among the top five specialties across all durations of OCS use. Pediatrics and otolaryngology were among the top five specialties for overall OCS use as well as short‐ and medium‐term use. Rheumatology and pulmonology were among the top five specialties for long‐term use, prescribing 32.4% and 6.7% of long‐term prescriptions, respectively. The annual prescribing frequencies of the top five physician specialties for OCS use during 2009–2018 are presented in Tables [Link], [Link]. Overall, OCS prescribing by family practice physicians remained stable in the United States, but declined in Taiwan; prescribing by internal medicine physicians increased in the United States but declined in Taiwan. Prescribing by pediatricians declined in both countries. Similar trends were seen across all time periods. In Taiwan, short‐ and medium‐term prescribing by dermatologists in Taiwan increased, and prescribing by otolaryngologists increased across all durations. In both countries, long‐term prescribing by rheumatologists decreased and long‐term prescribing by pulmonologists increased.

**TABLE 3:** Top five physician specialties of overall, short‐, medium‐ and long‐term oral corticosteroid use in the United States and Taiwan.

USA		Taiwan	
Top 5 physician specialties	%	Top 5 physician specialties	%
Overall use			
Family practice	31.6	Dermatology	22.0
Internal medicine	15.8	Family practice	19.3
Emergency medicine	8.2	Otolaryngology	14.8
Pediatrics	6.7	Internal medicine	11.8
Rheumatology	4.6	Pediatrics	11.2
Short‐term use			
Family practice	34.7	Dermatology	25.8
Internal medicine	15.7	Family practice	20.4
Emergency medicine	9.5	Otolaryngology	18.3
Pediatrics	8.0	Pediatrics	12.0
Surgery	4.9	Internal medicine	11.7
Medium‐term use			
Family practice	23.8	Family practice	22.6
Internal medicine	17.3	Dermatology	19.4
Rheumatology	8.6	Internal medicine	13.5
Pulmonology	5.8	Pediatrics	12.7
Otolaryngology	4.8	Otolaryngology	11.3
Long‐term use			
Rheumatology	31.6	Rheumatology	32.4
Internal medicine	15.0	Family practice	10.7
Family practice	13.8	Internal medicine	10.2
Pulmonology	5.8	Dermatology	7.3
Gastroenterology	3.3	Pulmonology	6.7

In the United States, non‐physician providers, such as physician assistants and nurse practitioners, were responsible for 16.0% of OCS prescriptions over the study period (Table S8). The annual frequency of OCS prescribed by these prescribers increased over time, from 6.0% in 2009 to 23.3% in 2018. These statistics are not available for Taiwan.

## DISCUSSION

Our study has four key findings. First, in this international population‐based longitudinal cohort, the average annual prevalence of overall OCS use in the general population varied substantially between countries during 2009–2018, at 6.8% in the United States, 17.5% in Taiwan, and 2.2% in Denmark. Second, the prevalence of OCS use in each of these three countries increased during the 10‐year study period: from 6.4% to 7.7% in the United States, from 16.6% to 18.7% in Taiwan, and from 1.7% to 2.9% in Denmark. This rise was observed only among adults and predominantly driven by increased short‐term OCS use. Third, in the United States and Taiwan, one‐third to one‐half of OCS users were healthy adults without baseline comorbid conditions, and a fifth to a quarter of OCS prescriptions were associated with a diagnosis of self‐limited respiratory infection, for which corticosteroids are known to be of limited benefit.^24^, ^25^, ^26^ Fourth, family practice and internal medicine physicians were among the highest OCS prescribers in these countries.

It is important to contextualize the clinical significance of the noted international increase in OCS prescribing over the study period. The percentage increases noted in the United States, Taiwan, and Denmark with an approximately additional 346,800, 471,700, and 65,800 patients in each country, respectively, in 2018 relative to 2009. Our evidence‐based, real‐world data suggest that the majority of increased OCS prescribing over the past decade is due to short‐term use by healthy, working‐age adults. This is the first study to demonstrate a longitudinal increase in OCS prescribing among healthy adults internationally, and underscores the need for judicious use of OCS. Recent work has demonstrated significant associations between short‐term OCS use and serious harms, such as gastrointestinal bleeding, heart failure, serious infection, venous thromboembolism, and fracture.^6^, ^9^, ^28^ As these risks persist in young, otherwise healthy individuals, the population‐level harms of short‐term OCS exposure remain a critical concern even when clinicians avoid prescribing to elderly or comorbid “high‐risk” patients.

These harms are particularly important to consider as OCS are commonly prescribed for self‐limited conditions (e.g., in this study, bronchitis and upper respiratory infections) and when treatment benefit is unclear. Particularly, we found that OCS prescriptions in the United States and Taiwan are commonly administered to patients with respiratory infections, allergic reactions, skin disorders, and back problems, all of which are typically self‐limited conditions with effective non‐steroidal treatments. These data provide important global context to recent studies evaluating risks attributable to avoidable corticosteroid prescriptions.^6^, ^8^, ^9^ Similar to successful initiatives to optimize opioid and antibiotic prescriptions, implementing a model of “corticosteroid stewardship” may facilitate reducing excess harms related to avoidable OCS use.^5^, ^29^

We noted substantial international variation in OCS use. In Denmark, one in 50 individuals on average received an OCS prescription each year, versus one in 14 in the United States and one in six in Taiwan. Several possible explanations are proposed for the observed differences. First, respiratory infection was among the top 10 indications for OCS use in the United States and Taiwan, but not in Denmark. This suggests that physicians in Denmark may prescribe OCS less commonly to patients with such self‐limited conditions. Second, prescriptions for OCS in the United States had, on average, higher doses and longer durations than those in Taiwan. Third, specialists were responsible for 70% of physician‐prescribed OCS in Taiwan, but only 53% in the United States. These observed differences may reflect different payer mechanisms for specialist care in the United States and Taiwan; specialist care requires a referral and is reimbursed at a higher rate in the United States, but is accessible to all residents at the same cost as non‐specialist care in Taiwan. The observed differences across the three countries should be interpreted with caution. Additional studies to elucidate mechanisms of international variation in OCS use are warranted.

As opposed to Taiwan and Denmark, OCS can be prescribed by mid‐level providers in the United States. We noted that such prescribing increased almost fivefold during the study period, with OCS prescriptions written by mid‐levels accounting for nearly a tenth of all OCS prescriptions in 2018. Potential contributors include the rise in mid‐level credentialing and utilization during this time period and different prescribing practices among mid‐level providers versus physicians.^29^, ^30^, ^31^ It is unknown whether the rise in OCS prescribing is attributable to mid‐levels acting as generalists (e.g., in internal medicine or family practice clinics) or as specialists (e.g., in dermatology, gastroenterology, or rheumatology clinics). Additional work to clarify the impact of mid‐level prescribing on increasing OCS use in the United States is needed.

Our findings regarding long‐term OCS use are in line with previous studies.^14^, ^15^, ^16^, ^17^, ^18^, ^19^ In both the United States and Taiwan, osteoarthritis and other non‐traumatic joint disorders were the most common indications for long‐term OCS prescriptions. This categorization includes rheumatic diseases, such as inflammatory arthritis. In both the United States and Taiwan, rheumatologists were the specialists most commonly prescribing long‐term OCS. However, non‐specialist physicians were also responsible for a considerable proportion of long‐term OCS; approximately one‐third of prescriptions in the United States and one‐fifth in Taiwan. Consistent with prior studies, we also observed that long‐term OCS use was more prevalent among women than men.^15^, ^16^, ^17^, ^18^, ^19^

Several strengths of this study should be noted. This is the first international study using population‐level claims data to compare and contrast OCS prescribing patterns over the past decade. It is one of only a few recent studies evaluating longitudinal trends across different durations of OCS use in the general population, as most previous studies used cross‐sectional data to assess prevalence of only long‐term use. Limitations of this work include differences in data features which prevent us from comparing certain information across countries, the notable trends observed among mid‐level providers in the United States, which are not applicable to Taiwan and Denmark, demographic differences related to the use of population‐level medical claims data in Taiwan and Denmark versus commercial claims data in the United States (which may not adequately capture populations eligible for government programs such as Medicare and Medicaid), and the lack of data available for mid‐ and low income countries, which limits generalizability.^32^ Although we know of no data to suggest this, it may be theoretically possible that population‐level differences in OCS metabolism might influence response to treatment and, ultimately, prescribing patterns.

This international population‐based longitudinal cohort study provides real‐world evidence of an ongoing steady increase in OCS use in the general populations of the United States, Taiwan, and Denmark. The rise seen over the past decade was largely driven by short‐term OCS prescribing to healthy adults. This study lends further support to call for a model of “corticosteroid stewardship” when prescribing OCS, particularly as in regard to the judicious use of OCS to treat healthy people for self‐limited conditions. It is important to implement effective strategies to prevent avoidable harms caused by OCS use.

## AUTHOR CONTRIBUTIONS

B.I.W., T.‐C.Y., and H.‐J.T. wrote the manuscript. B.I.W., H.‐J.T., K.A., C.T.‐P., A.K.W., and T.‐C.Y. designed the research. P.L., K.A., and Y.‐F.T. performed the research. B.I.W., H.‐J.T., K.A., A.C.W., A.K.W., and T.‐C.Y. analyzed the data.

## FUNDING INFORMATION

This work was supported by grants from National Health Research Institutes, Taiwan (PI: Tsai, PH‐111‐PP‐08), Ministry of Science and Technology of Taiwan (PIs: Tsai, MOST 107‐2314‐B‐400‐031‐MY3; and Yao, MOST 106‐2314‐B‐182‐051‐MY3 and MOST 109‐2314‐B‐182‐042‐MY3), Chang Gung Medical Foundation (PI: Yao, CMRPG3F1711‐3, CMRPG3F0361, CMRPG3J0121, and CMRPG3K1371). Dr. Wallace received no direct support for this work, but her effort during the time of this work was supported by the Veterans Affairs Administration (IK2CX002430) and the National Center for Advancing Translational Sciences (KL2TR002241).

## CONFLICT OF INTEREST STATEMENT

The authors declared no competing interests for this work.

## Supporting information


Figure S1

Figure S2

Figure S3

Figure S4

Table S1

Table S2

Table S3

Table S4

Table S5

Table S6

Table S7

Table S8
