# Supplementary material for: Prevalence and prescribing patterns of oral corticosteroids in the United States, Taiwan, and Denmark, 2009–2018
Source: Clin Transl Sci. 2023 Oct 6;16(12):2565–76. doi: 10.1111/cts.13649 (PMC10719491; doi:10.1111/cts.13649)
Supplement: Supplementary file 1 — Figure S1 [file CTS-16-2565-s010.pdf]

Figure S1. Flow chart of study participants.

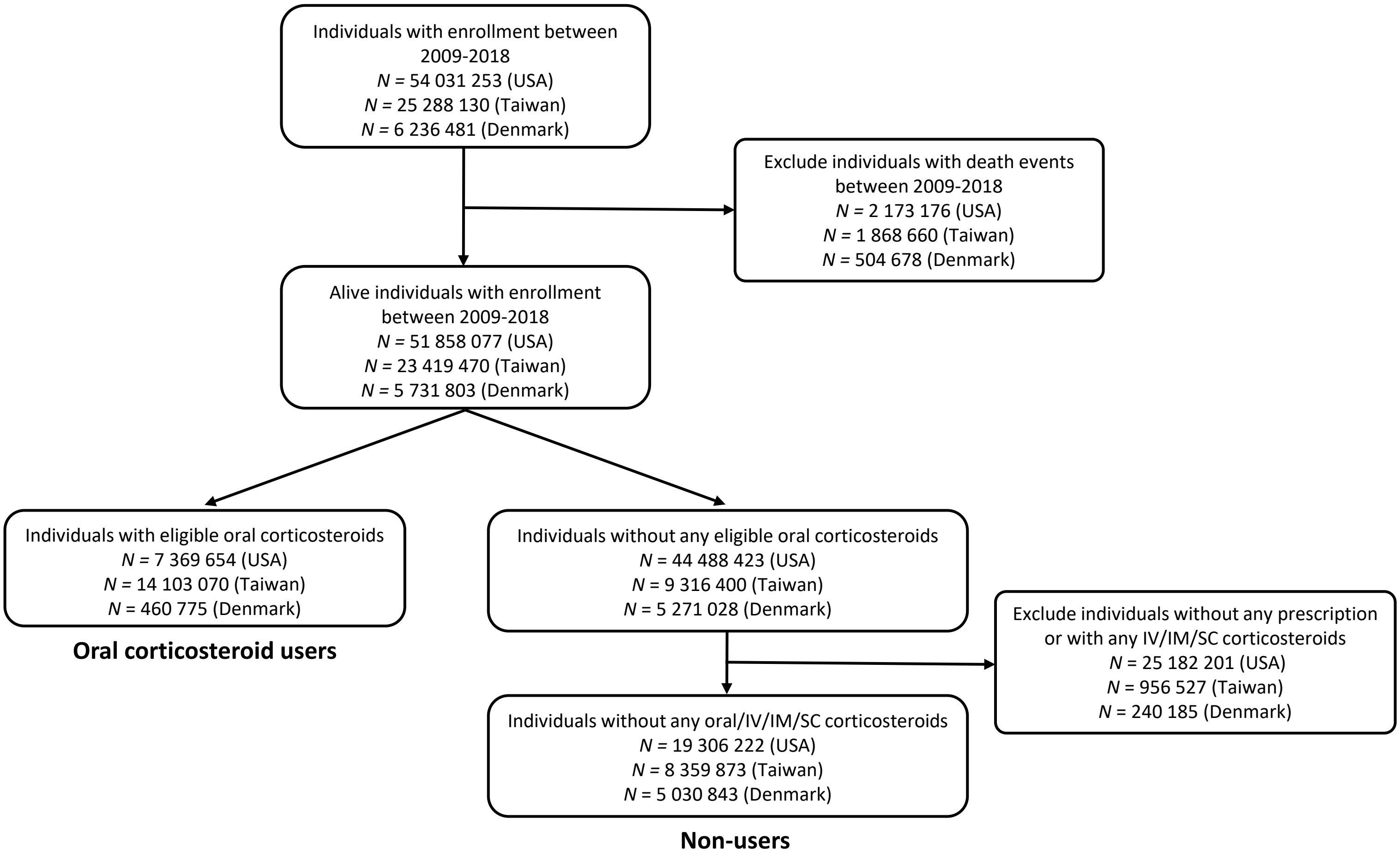

Abbreviation: **IV**: intravenous; **IM**: intramuscular; **SC**: subcutaneous.
