# Supplementary material for: Prevalence and prescribing patterns of oral corticosteroids in the United States, Taiwan, and Denmark, 2009–2018
Source: Clin Transl Sci. 2023 Oct 6;16(12):2565–76. doi: 10.1111/cts.13649 (PMC10719491; doi:10.1111/cts.13649)
Supplement: Supplementary file 2 — Figure S2 [file CTS-16-2565-s008.pdf]

Figure S2. Ten-year trend on prevalence of overall, short-, medium-, and long-term OCS use in USA (**A**) and Taiwan (**B**)

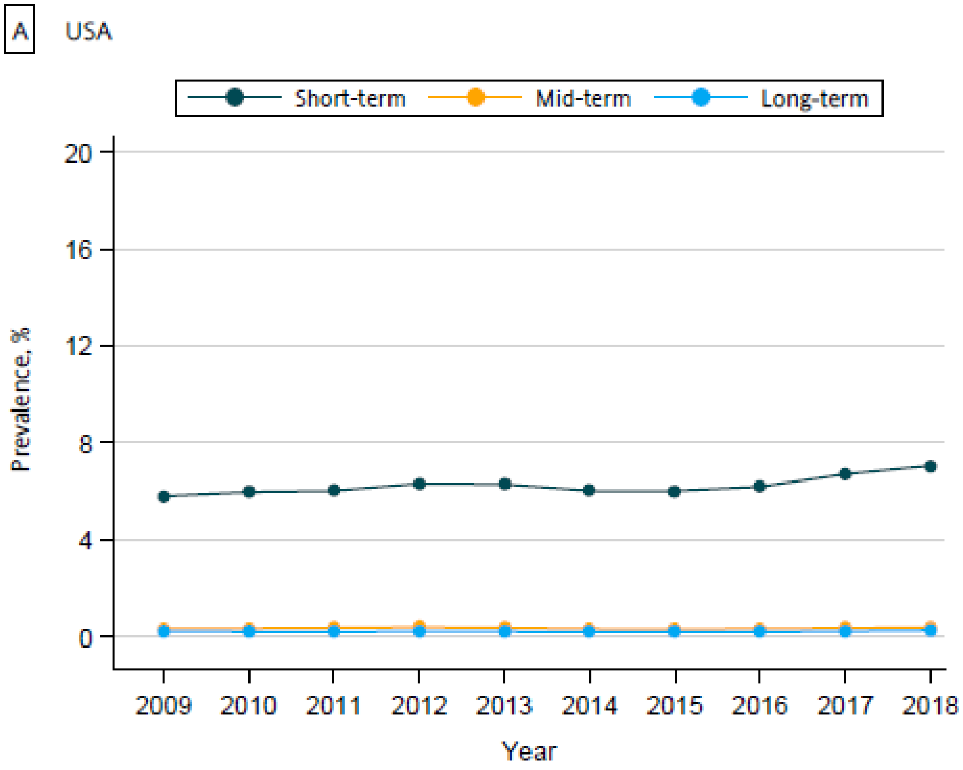

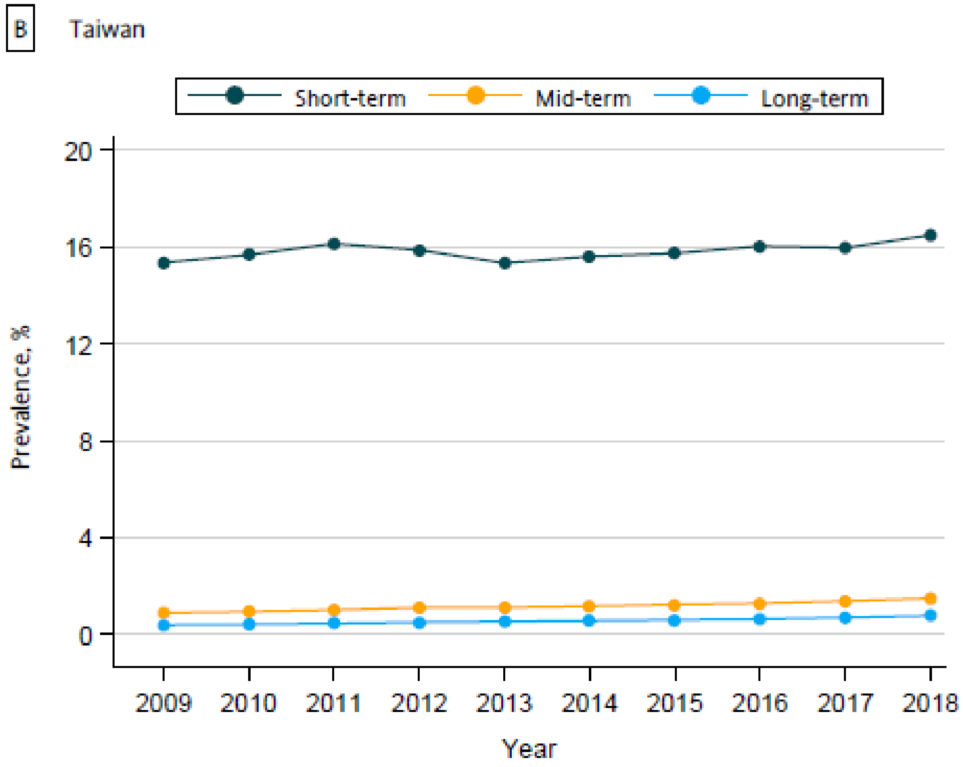

($P_{\text{linear trend}}$=0.02, 0.008, 0.97, and 0.32 for overall, short-, medium-, and long-term in USA; $P_{\text{linear trend}}$=0.001, 0.07, <.0001, and <.0001 for overall, short-, medium-, and long-term in Taiwan).
