# Supplementary material for: Prevalence and prescribing patterns of oral corticosteroids in the United States, Taiwan, and Denmark, 2009–2018
Source: Clin Transl Sci. 2023 Oct 6;16(12):2565–76. doi: 10.1111/cts.13649 (PMC10719491; doi:10.1111/cts.13649)
Supplement: Supplementary file 3 — Figure S3 [file CTS-16-2565-s003.pdf]

Figure S3. Ten-year trend on prevalence of short-, medium-, and long-term OCS use in USA (**A-C**) and Taiwan (**D-F**), stratified by age

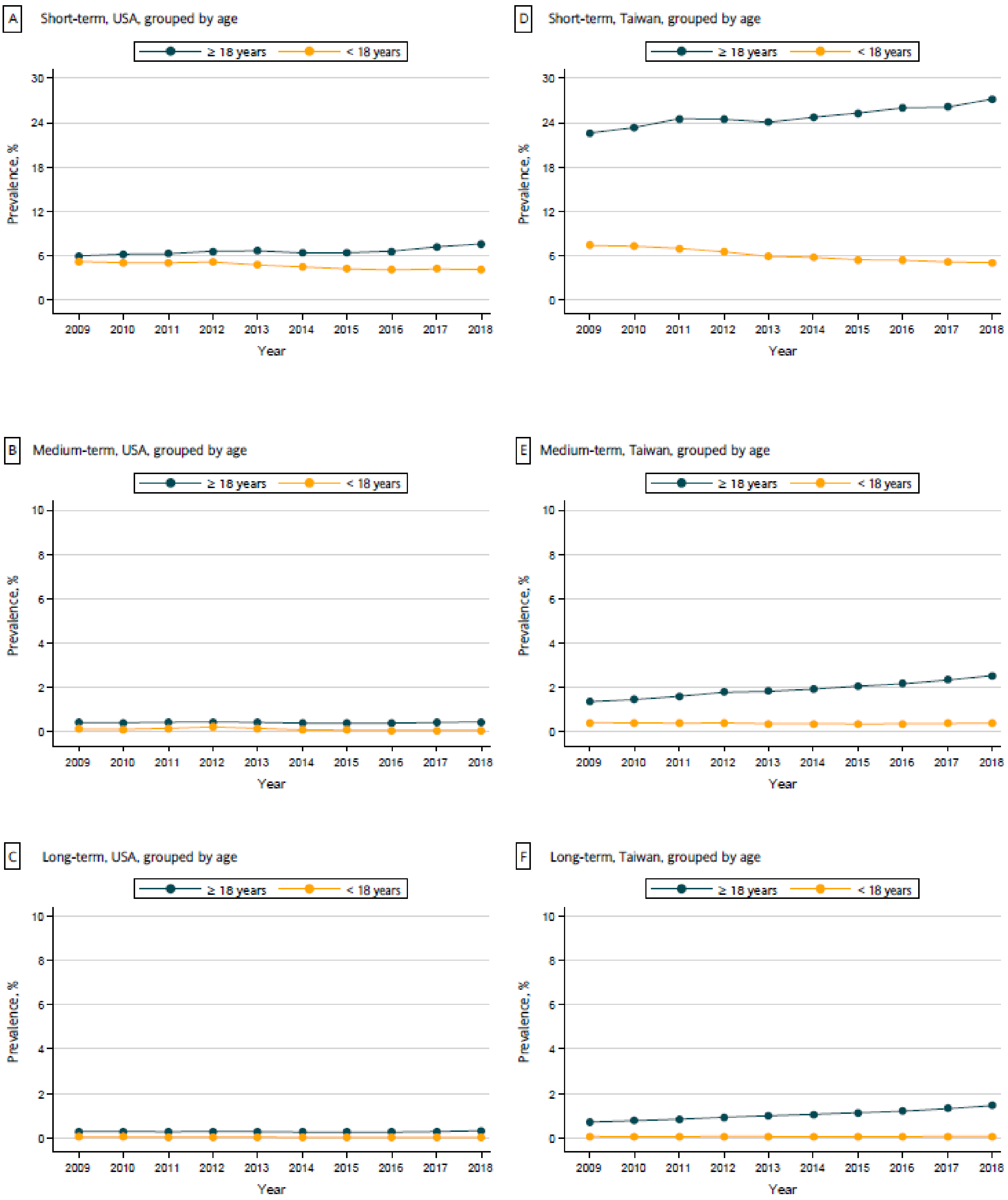

$P_{\text{linear trend}}$=0.002, 0.82, and 0.66 for short-, medium-, and long-term for individuals age ≧18 years in USA; $P_{\text{linear trend}}$=<.0001, <.0001, and <.0001 for short-, medium-, and long-term for individuals age ≧18 years in Taiwan; $P_{\text{linear trend}}$=<.0001, 0.04, and 0.001 for short-, medium-, and long-term for individuals age <18 years in USA; $P_{\text{linear trend}}$=<.0001, 0.04, and 0.70 for short-, medium-, and long-term for individuals age <18 years in Taiwan.
