# Supplementary material for: Prevalence and prescribing patterns of oral corticosteroids in the United States, Taiwan, and Denmark, 2009–2018
Source: Clin Transl Sci. 2023 Oct 6;16(12):2565–76. doi: 10.1111/cts.13649 (PMC10719491; doi:10.1111/cts.13649)
Supplement: Supplementary file 4 — Figure S4 [file CTS-16-2565-s005.pdf]

Figure S4. Ten-year trend on prevalence of short-, medium-, and long-term OCS use in USA (**A-C**) and Taiwan (**D-F**), stratified by sex

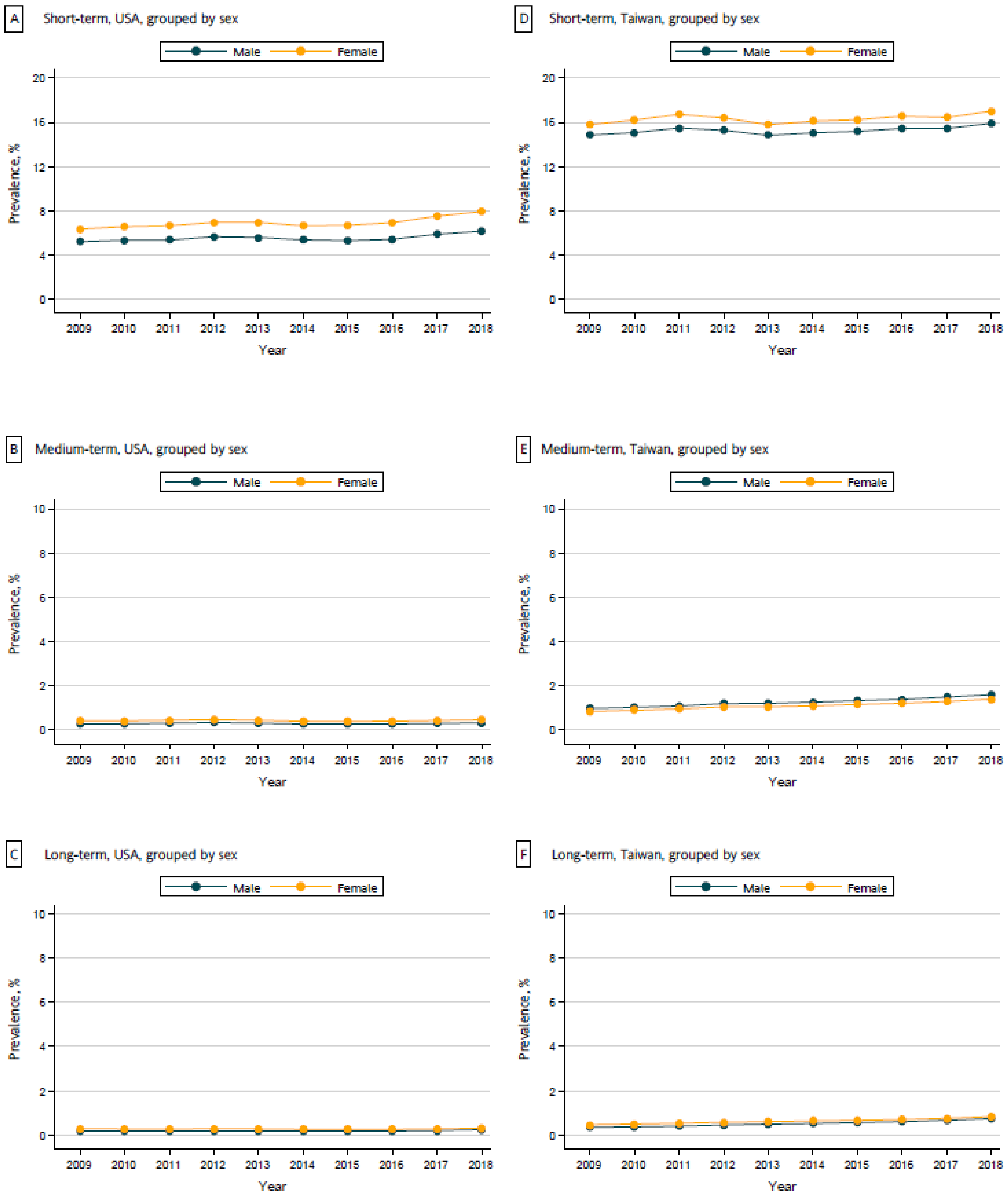

$P_{\_\text{linear trend}}$=0.02, 0.92, and 1.00 for short-, medium-, and long-term for males in USA; $P_{\_\text{linear trend}}$=0.04, <.0001, and <.0001 for short-, medium-, and long-term for males in Taiwan; $P_{\_\text{linear trend}}$=0.004, 0.91, and 0.07 for short-, medium-, and long-term for females in USA; $P_{\_\text{linear trend}}$=0.10, <.0001, and <.0001 for short-, medium-, and long-term for females in Taiwan.
