# Supplementary material for: Prevalence and prescribing patterns of oral corticosteroids in the United States, Taiwan, and Denmark, 2009–2018
Source: Clin Transl Sci. 2023 Oct 6;16(12):2565–76. doi: 10.1111/cts.13649 (PMC10719491; doi:10.1111/cts.13649)
Supplement: Supplementary file 5 — Table S1 [file CTS-16-2565-s001.docx]

**Table S1.** Equivalent doses of oral corticosteroids investigated in this study.

| **Corticosteroids** | **Equivalent Dose** | |
| --- | --- | --- |
| Betamethasone | 0.6 | mg |
| Dexamethasone | 0.75 | mg |
| Methylprednisolone | 4 | mg |
| Triamcinolone | 4 | mg |
| Prednisone | 5 | mg |
| Prednisolone | 5 | mg |
| Hydrocortisone | 20 | mg |
| Cortisone | 25 | mg |
