# Supplementary material for: Prevalence and prescribing patterns of oral corticosteroids in the United States, Taiwan, and Denmark, 2009–2018
Source: Clin Transl Sci. 2023 Oct 6;16(12):2565–76. doi: 10.1111/cts.13649 (PMC10719491; doi:10.1111/cts.13649)
Supplement: Supplementary file 6 — Table S2 [file CTS-16-2565-s011.docx]

**Table S2.** Overall durations and doses of short-, medium- and long-term oral corticosteroids use over the 10 year study period in USA and Taiwan.

|  | **USA** | **Taiwan** |
| --- | --- | --- |
|  | **Short-term use**^c^ | |
| Median (IQR) daily dose (mg/day)^a^ | 20.0 (17.5-39.2) | 11.7 (8.3-15.0) |
| Median (IQR) duration per year (days)^b^ | 6.0 (5.0-10.0) | 4.7 (3.0-7.2) |
| Median (IQR) cumulative dose per year | 160.0 (105.0-280.0) | 53.8 (36.0-90.0) |
|  | **Medium-term use**^c^ | |
| Median (IQR) daily dose (mg/day) | 20.0 (10.9-30.0) | 6.3 (3.4-10.3) |
| Median (IQR) duration per year (days) | 38.0 (30.0-55.0) | 42.0 (34.0-56.0) |
| Median (IQR) cumulative dose per year | 820.0 (480.0-1275.0) | 288.0 (140.0-480.0) |
|  | **Long-term use**^c^ | |
| Median (IQR) daily dose (mg/day) | 9.3 (5.0-15.7) | 6.9 (4.5-10.3) |
| Median (IQR) duration per year (days) | 200.0 (125.0-310.0) | 137.3 (112.0-182.0) |
| Median (IQR) cumulative dose per year | 1820.0 (1130.0-3010.0) | 1026.0 (595.0-1647.6) |

Note:

^a^ Dose reported in prednisone equivalents.

^b^ Divided by the year of actual oral corticosteroids use instead of total study year.

^c^ Short-term corticosteroids use: 1-29 days per year; mid-term corticosteroids use: 30-89 days per year; long-term corticosteroids use: >=90 days per year.
