# Supplementary material for: Prevalence and prescribing patterns of oral corticosteroids in the United States, Taiwan, and Denmark, 2009–2018
Source: Clin Transl Sci. 2023 Oct 6;16(12):2565–76. doi: 10.1111/cts.13649 (PMC10719491; doi:10.1111/cts.13649)
Supplement: Supplementary file 7 — Table S3 [file CTS-16-2565-s006.docx]

**Table S3.** Frequency of each prescribed oral corticosteroids in USA, Taiwan, and Denmark.

| **Corticosteroids, *n* (%)** | **USA** | **Taiwan** | **Denmark** |
| --- | --- | --- | --- |
| Betamethasone | 0 | 6 906 929 (8.1%) | 357 262 (9.3%) |
| Dexamethasone | 609 991 (3.7%) | 19 758 892 (23.1%) | 1 914 (0.1%) |
| Methylprednisolone | 5 100 693 (31.3%) | 10 755 117 (12.5%) | 293 351 (7.7%) |
| Triamcinolone | 0 | 952 811 (1.1%) | 0 |
| Prednisone | 9 263 576 (56.8%) | 0 | 142 297 (3.7%) |
| Prednisolone | 1 156 670 (7.1%) | 46 585 656 (54.4%) | 2 862 491 (74.7%) |
| Hydrocortisone | 176 102 (1.1%) | 0 | 80 437 (2.1%) |
| Cortisone | 2827 (0.0%) | 696 712 (0.8%) | 0 |
