# Supplementary material for: Prevalence and prescribing patterns of oral corticosteroids in the United States, Taiwan, and Denmark, 2009–2018
Source: Clin Transl Sci. 2023 Oct 6;16(12):2565–76. doi: 10.1111/cts.13649 (PMC10719491; doi:10.1111/cts.13649)
Supplement: Supplementary file 8 — Table S4 [file CTS-16-2565-s009.docx]

| **Table S4a.** Trend of top 10 indications and top 5 physician specialties of overall oral corticosteroids use from 2009-2018 in the USA | | | | | | | | | | | | | | | | | | | | | | |
| --- | --- | --- | --- | --- | --- | --- | --- | --- | --- | --- | --- | --- | --- | --- | --- | --- | --- | --- | --- | --- | --- | --- |
|  | **Overall** | | **2009** | | **2010** | | **2011** | | **2012** | | **2013** | | **2014** | | **2015** | | **2016** | | **2017** | | **2018** | |
|  | ***n (%)*** | ***Rank*** | ***n (%)*** | ***Rank*** | ***n (%)*** | ***Rank*** | ***n (%)*** | ***Rank*** | ***n (%)*** | ***Rank*** | ***n (%)*** | ***Rank*** | ***n (%)*** | ***Rank*** | ***n (%)*** | ***Rank*** | ***n (%)*** | ***Rank*** | ***n (%)*** | ***Rank*** | ***n (%)*** | ***Rank*** |
| **Top 10 indications, *n* (%)** | | | | | | | | | | | | | | | | | | | | | | |
| Acute Bronchitis and URI | 2929243 (18.7%) | 1 | 239820 (18.1%) | 1 | 238976 (18.7%) | 1 | 243611 (18.7%) | 1 | 262619 (18.6%) | 1 | 262183 (18.3%) | 1 | 237992 (17.7%) | 1 | 277271 (18.4%) | 1 | 341693 (19.6%) | 1 | 404304 (19.8%) | 1 | 420774 (18.7%) | 1 |
| COPD, Asthma, and Other Respiratory Conditions | 2553734 (16.3%) | 2 | 228794 (17.3%) | 2 | 218345 (17.0%) | 2 | 218580 (16.7%) | 2 | 237136 (16.8%) | 2 | 240814 (16.8%) | 2 | 232043 (17.2%) | 2 | 249855 (16.5%) | 2 | 263092 (15.1%) | 2 | 316467 (15.5%) | 2 | 348608 (15.5%) | 2 |
| Back Problems | 1141576 (7.3%) | 3 | 85772 (6.5%) | 4 | 89538 (7.0%) | 4 | 93945 (7.2%) | 4 | 103603 (7.3%) | 3 | 107044 (7.5%) | 3 | 104051 (7.7%) | 3 | 114470 (7.6%) | 3 | 129474 (7.4%) | 4 | 149744 (7.3%) | 4 | 163935 (7.3%) | 4 |
| Allergic Reactions | 945300 (6.0%) | 4 | 100776 (7.6%) | 3 | 99720 (7.8%) | 3 | 99118 (7.6%) | 3 | 102634 (7.3%) | 4 | 99721 (7.0%) | 4 | 89321 (6.6%) | 4 | 89647 (5.9%) | 4 | 81643 (4.7%) | 6 | 89127 (4.4%) | 6 | 93593 (4.2%) | 6 |
| Osteoarthritis and Other Non-Traumatic Joint Disorders | 918292 (5.9%) | 5 | 57982 (4.4%) | 5 | 57548 (4.5%) | 5 | 60743 (4.7%) | 5 | 68024 (4.8%) | 5 | 70904 (4.9%) | 5 | 67204 (5.0%) | 5 | 84625 (5.6%) | 5 | 129863 (7.5%) | 3 | 151352 (7.4%) | 3 | 170047 (7.6%) | 3 |
| Skin Disorders | 586185 (3.7%) | 6 | 40438 (3.1%) | 7 | 40873 (3.2%) | 7 | 41535 (3.2%) | 7 | 45381 (3.2%) | 7 | 45320 (3.2%) | 7 | 43378 (3.2%) | 7 | 50610 (3.4%) | 7 | 83533 (4.8%) | 5 | 93779 (4.6%) | 5 | 101338 (4.5%) | 5 |
| Systemic Lupus and Connective Tissue Disorders | 506744 (3.2%) | 7 | 51097 (3.9%) | 6 | 50045 (3.9%) | 6 | 51248 (3.9%) | 6 | 57481 (4.1%) | 6 | 58405 (4.1%) | 6 | 55119 (4.1%) | 6 | 54614 (3.6%) | 6 | 38936 (2.2%) | 8 | 43178 (2.1%) | 9 | 46621 (2.1%) | 10 |
| Nervous System Disorders | 441442 (2.8%) | 8 | 33624 (2.5%) | 8 | 33743 (2.6%) | 8 | 35181 (2.7%) | 8 | 37863 (2.7%) | 8 | 39039 (2.7%) | 8 | 36739 (2.7%) | 8 | 41768 (2.8%) | 8 | 52274 (3.0%) | 7 | 61367 (3.0%) | 7 | 69844 (3.1%) | 7 |
| Trauma-Related Disorders | 319686 (2.0%) | 9 | 27842 (2.1%) | 9 | 27996 (2.2%) | 9 | 28415 (2.2%) | 9 | 31124 (2.2%) | 9 | 31995 (2.2%) | 9 | 30371 (2.3%) | 9 | 32650 (2.2%) | 9 | 32997 (1.9%) | 10 | 36625 (1.8%) | 10 | 39671 (1.8%) | 13 |
| Hypertension | 291820 (1.9%) | 10 | 20929 (1.6%) | 11 | 21649 (1.7%) | 11 | 21950 (1.7%) | 12 | 24225 (1.7%) | 12 | 25351 (1.8%) | 11 | 23031 (1.7%) | 11 | 26913 (1.8%) | 10 | 33893 (1.9%) | 9 | 43415 (2.1%) | 8 | 50464 (2.2%) | 9 |
| **Top 5 physician specialties, *n* (%)** | | | | | | | | | | | | | | | | | | | | | | |
| Family Practice | 3646227 (31.6%) | 1 | 330103 (30.2%) | 1 | 329963 (31.3%) | 1 | 330527 (31.3%) | 1 | 355180 (31.6%) | 1 | 350011 (31.4%) | 1 | 320028 (31.7%) | 1 | 347053 (31.8%) | 1 | 390065 (32.1%) | 1 | 440608 (32.4%) | 1 | 452689 (31.9%) | 1 |
| Internal Medicine | 1819504 (15.8%) | 2 | 161335 (14.8%) | 2 | 159805 (15.2%) | 2 | 160878 (15.2%) | 2 | 173040 (15.4%) | 2 | 173334 (15.6%) | 2 | 155557 (15.4%) | 2 | 173662 (15.9%) | 2 | 197331 (16.3%) | 2 | 226773 (16.7%) | 2 | 237789 (16.8%) | 2 |
| Emergency Medicine | 949565 (8.2%) | 3 | 78631 (7.2%) | 4 | 76234 (7.2%) | 4 | 80952 (7.7%) | 4 | 91526 (8.1%) | 3 | 92251 (8.3%) | 3 | 88580 (8.8%) | 3 | 96268 (8.8%) | 3 | 107402 (8.8%) | 3 | 119417 (8.8%) | 3 | 118304 (8.3%) | 3 |
| Pediatrics | 773048 (6.7%) | 4 | 107712 (9.9%) | 3 | 94379 (9.0%) | 3 | 90920 (8.6%) | 3 | 88638 (7.9%) | 4 | 77877 (7.0%) | 4 | 66051 (6.6%) | 4 | 62914 (5.8%) | 4 | 61555 (5.1%) | 4 | 63374 (4.7%) | 4 | 59628 (4.2%) | 6 |
| Rheumatology | 529352 (4.6%) | 5 | 50824 (4.7%) | 5 | 44956 (4.3%) | 7 | 45800 (4.3%) | 7 | 49807 (4.4%) | 6 | 51060 (4.6%) | 5 | 46628 (4.6%) | 5 | 51456 (4.7%) | 5 | 57611 (4.7%) | 5 | 62771 (4.6%) | 5 | 68439 (4.8%) | 4 |
|  |  |  |  |  |  |  |  |  |  |  |  |  |  |  |  |  |  |  |  |  |  |  |
| Abbreviation: **URI**: upper respiratory infection; **COPD**: chronic obstructive pulmonary disease. | | | | | | | | | | | | | | | | | | | | | | |

| **Table S4b.** Trend of top 10 indications and top 5 physician specialties of overall oral corticosteroids use from 2009-2018 in Taiwan | | | | | | | | | | | | | | | | | | | | | | |
| --- | --- | --- | --- | --- | --- | --- | --- | --- | --- | --- | --- | --- | --- | --- | --- | --- | --- | --- | --- | --- | --- | --- |
|  | **Overall** | | **2009** | | **2010** | | **2011** | | **2012** | | **2013** | | **2014** | | **2015** | | **2016** | | **2017** | | **2018** | |
|  | ***n (%)*** | ***Rank*** | ***n (%)*** | ***Rank*** | ***n (%)*** | ***Rank*** | ***n (%)*** | ***Rank*** | ***n (%)*** | ***Rank*** | ***n (%)*** | ***Rank*** | ***n (%)*** | ***Rank*** | ***n (%)*** | ***Rank*** | ***n (%)*** | ***Rank*** | ***n (%)*** | ***Rank*** | ***n (%)*** | ***Rank*** |
| **Top 10 indications, *n* (%)** | | | | | | | | | | | | | | | | | | | | | | |
| Acute Bronchitis and URI | 21478021 (24.8%) | 1 | 2009017 (27.3%) | 1 | 2122791 (27.6%) | 1 | 2388272 (29.1%) | 1 | 2249111 (26.8%) | 1 | 2010579 (24.5%) | 1 | 2084364 (24.4%) | 1 | 1996165 (22.8%) | 1 | 2199664 (24.0%) | 1 | 2127411 (22.7%) | 1 | 2290605 (23.0%) | 1 |
| Allergic Reactions | 16776870 (19.6%) | 2 | 1528448 (20.8%) | 2 | 1596241 (20.7%) | 2 | 1624273 (19.8%) | 2 | 1674894 (20.0%) | 2 | 1735061 (21.2%) | 2 | 1812500 (21.2%) | 2 | 1919765 (21.9%) | 2 | 1602176 (17.5%) | 2 | 1628346 (17.4%) | 2 | 1654968 (16.6%) | 2 |
| COPD, Asthma, and Other Respiratory Conditions | 10635469 (12.4%) | 3 | 931223 (12.7%) | 3 | 968435 (12.6%) | 3 | 1025736 (12.5%) | 3 | 1066969 (12.7%) | 3 | 1029969 (12.6%) | 3 | 1068546 (12.5%) | 3 | 1085039 (12.4%) | 3 | 1103053 (12.0%) | 4 | 1141466 (12.2%) | 4 | 1214589 (12.2%) | 4 |
| Skin Disorders | 8869350 (10.4%) | 4 | 673957 (9.2%) | 4 | 676313 (8.8%) | 4 | 689866 (8.4%) | 4 | 728278 (8.7%) | 4 | 732793 (8.9%) | 4 | 759850 (8.9%) | 4 | 841647 (9.6%) | 4 | 1213505 (13.2%) | 3 | 1265903 (13.5%) | 3 | 1286800 (12.9%) | 3 |
| Osteoarthritis and Other Non-Traumatic Joint Disorders | 4419078 (5.2%) | 5 | 320712 (4.4%) | 5 | 334011 (4.3%) | 5 | 358646 (4.4%) | 5 | 398955 (4.8%) | 5 | 410107 (5.0%) | 5 | 419795 (4.9%) | 5 | 440248 (5.0%) | 5 | 556741 (6.1%) | 5 | 579131 (6.2%) | 5 | 598944 (6.0%) | 5 |
| Systemic Lupus and Connective Tissue Disorders | 2908362 (3.4%) | 6 | 252241 (3.4%) | 6 | 276314 (3.6%) | 6 | 295565 (3.6%) | 6 | 323453 (3.9%) | 6 | 324482 (4.0%) | 6 | 340482 (4.0%) | 6 | 355254 (4.1%) | 6 | 234390 (2.6%) | 6 | 249374 (2.7%) | 6 | 256400 (2.6%) | 7 |
| Tonsillitis | 2111878 (2.4%) | 7 | 168842 (2.3%) | 9 | 168801 (2.2%) | 9 | 194133 (2.4%) | 8 | 201967 (2.4%) | 8 | 204978 (2.5%) | 8 | 221517 (2.6%) | 8 | 204616 (2.3%) | 9 | 231451 (2.5%) | 7 | 238489 (2.5%) | 7 | 277080 (2.8%) | 6 |
| Trauma-Related Disorders | 1477072 (0.8%) | 8 | 176609 (2.4%) | 7 | 197370 (2.6%) | 7 | 204721 (2.5%) | 7 | 217639 (2.6%) | 7 | 217827 (2.7%) | 7 | 225719 (2.6%) | 7 | 231375 (2.6%) | 7 | 218431 (2.4%) | 8 | 215737 (2.3%) | 8 | 245521 (2.5%) | 8 |
| Infectious Diseases | 1378351 (1.1%) | 9 | 172998 (2.4%) | 8 | 181802 (2.4%) | 8 | 187049 (2.3%) | 9 | 196198 (2.3%) | 9 | 201039 (2.5%) | 9 | 213387 (2.5%) | 9 | 224183 (2.6%) | 8 | 212083 (2.3%) | 9 | 215339 (2.3%) | 9 | 229744 (2.3%) | 9 |
| Back Problems | 1028752 (1.0%) | 10 | 134423 (1.8%) | 10 | 138184 (1.8%) | 10 | 148615 (1.8%) | 10 | 153636 (1.8%) | 10 | 149321 (1.8%) | 10 | 148925 (1.7%) | 12 | 155602 (1.8%) | 12 | 138784 (1.5%) | 12 | 150370 (1.6%) | 12 | 166671 (1.7%) | 12 |
| **Top 5 physician specialties, *n* (%)** | | | | | | | | | | | | | | | | | | | | | | |
| Dermatology | 18831575 (22.0%) | 1 | 1495196 (20.4%) | 2 | 1569488 (20.4%) | 2 | 1637469 (19.9%) | 2 | 1716200 (20.5%) | 2 | 1789860 (21.8%) | 1 | 1897296 (22.2%) | 1 | 2085968 (23.8%) | 1 | 2141715 (23.4%) | 1 | 2214937 (23.6%) | 1 | 2283446 (23.0%) | 1 |
| Family Practice | 16568428 (19.3%) | 2 | 1688633 (23.0%) | 1 | 1738303 (22.6%) | 1 | 1826467 (22.2%) | 1 | 1779877 (21.2%) | 1 | 1607947 (19.6%) | 2 | 1597493 (18.7%) | 2 | 1570065 (17.9%) | 2 | 1554632 (17.0%) | 2 | 1569834 (16.7%) | 2 | 1635177 (16.4%) | 2 |
| Otolaryngology | 12710062 (14.8%) | 3 | 1009433 (13.7%) | 3 | 1085020 (14.1%) | 3 | 1161488 (14.1%) | 3 | 1202377 (14.3%) | 3 | 1198918 (14.6%) | 3 | 1265328 (14.8%) | 3 | 1292107 (14.7%) | 3 | 1423233 (15.5%) | 3 | 1458168 (15.6%) | 3 | 1613990 (16.2%) | 3 |
| Internal Medicine | 10089243 (11.8%) | 4 | 975810 (13.3%) | 4 | 962500 (12.5%) | 4 | 1062150 (12.9%) | 4 | 1052415 (12.6%) | 4 | 999772 (12.2%) | 4 | 1024618 (12.0%) | 4 | 992052 (11.3%) | 4 | 1029922 (11.2%) | 4 | 980436 (10.5%) | 5 | 1009568 (10.1%) | 5 |
| Pediatrics | 9603517 (11.2%) | 5 | 869152 (11.8%) | 5 | 955360 (12.4%) | 5 | 1019665 (12.4%) | 5 | 988455 (11.8%) | 5 | 889865 (10.9%) | 5 | 958221 (11.2%) | 5 | 923995 (10.5%) | 5 | 972999 (10.6%) | 5 | 987700 (10.5%) | 4 | 1038105 (10.4%) | 4 |
| Note: |  |  |  |  |  |  |  |  |  |  |  |  |  |  |  |  |  |  |  |  |  |  |
| Abbreviation: **URI**: upper respiratory infection; **COPD**: chronic obstructive pulmonary disease. | | | | | | | | | | | | | | | | | | | | | | |
