# Supplementary material for: Prevalence and prescribing patterns of oral corticosteroids in the United States, Taiwan, and Denmark, 2009–2018
Source: Clin Transl Sci. 2023 Oct 6;16(12):2565–76. doi: 10.1111/cts.13649 (PMC10719491; doi:10.1111/cts.13649)
Supplement: Supplementary file 9 — Table S5 [file CTS-16-2565-s007.docx]

| **Table S5a.** Trend of top 10 indications and top 5 physician specialties of short-term oral corticosteroids use from 2009-2018 in the USA | | | | | | | | | | | | | | | | | | | | | | |
| --- | --- | --- | --- | --- | --- | --- | --- | --- | --- | --- | --- | --- | --- | --- | --- | --- | --- | --- | --- | --- | --- | --- |
|  | **Overall** | | **2009** | | **2010** | | **2011** | | **2012** | | **2013** | | **2014** | | **2015** | | **2016** | | **2017** | | **2018** | |
|  | ***n (%)*** | ***Rank*** | ***n (%)*** | ***Rank*** | ***n (%)*** | ***Rank*** | ***n (%)*** | ***Rank*** | ***n (%)*** | ***Rank*** | ***n (%)*** | ***Rank*** | ***n (%)*** | ***Rank*** | ***n (%)*** | ***Rank*** | ***n (%)*** | ***Rank*** | ***n (%)*** | ***Rank*** | ***n (%)*** | ***Rank*** |
| **Top 10 indications, *n* (%)** | | | | | | | | | | | | | | | | | | | | | | |
| Acute Bronchitis and URI | 2736935 (21.3%) | 1 | 222642 (20.7%) | 1 | 222714 (21.0%) | 1 | 225901 (21.1%) | 1 | 241821 (21.0%) | 1 | 244032 (20.7%) | 1 | 223734 (20.0%) | 1 | 260587 (20.9%) | 1 | 320902 (22.4%) | 1 | 379406 (22.7%) | 1 | 395196 (21.7%) | 1 |
| COPD, Asthma, and Other Respiratory Conditions | 2112008 (16.5%) | 2 | 192781 (17.9%) | 2 | 185370 (17.5%) | 2 | 183180 (17.1%) | 2 | 196592 (17.1%) | 2 | 201098 (17.1%) | 2 | 196643 (17.6%) | 2 | 207548 (16.7%) | 2 | 215464 (15.0%) | 2 | 255413 (15.3%) | 2 | 277919 (15.3%) | 2 |
| Back Problems | 1033363 (8.1%) | 3 | 77846 (7.2%) | 4 | 81802 (7.7%) | 4 | 85467 (8.0%) | 4 | 93464 (8.1%) | 3 | 97431 (8.3%) | 3 | 94894 (8.5%) | 3 | 103515 (8.3%) | 3 | 117192 (8.2%) | 3 | 134331 (8.0%) | 3 | 147421 (8.1%) | 3 |
| Allergic Reactions | 852344 (6.6%) | 4 | 91668 (8.5%) | 3 | 90830 (8.6%) | 3 | 89936 (8.4%) | 3 | 92634 (8.0%) | 4 | 90621 (7.7%) | 4 | 81591 (7.3%) | 4 | 80617 (6.5%) | 4 | 72711 (5.1%) | 5 | 79024 (4.7%) | 5 | 82712 (4.5%) | 6 |
| Osteoarthritis and Other Non-Traumatic Joint Disorders | 605570 (4.7%) | 5 | 31662 (2.9%) | 7 | 33958 (3.2%) | 6 | 35768 (3.3%) | 6 | 40353 (3.5%) | 5 | 43293 (3.7%) | 5 | 41624 (3.7%) | 5 | 55446 (4.5%) | 5 | 92891 (6.5%) | 4 | 108750 (6.5%) | 4 | 121825 (6.7%) | 4 |
| Skin Disorders | 481537 (3.8%) | 6 | 32626 (3.0%) | 6 | 33258 (3.1%) | 7 | 33729 (3.1%) | 7 | 36564 (3.2%) | 7 | 36750 (3.1%) | 7 | 35220 (3.2%) | 7 | 41341 (3.3%) | 6 | 70263 (4.9%) | 6 | 78284 (4.7%) | 6 | 83502 (4.6%) | 5 |
| Nervous System Disorders | 365060 (2.8%) | 7 | 27911 (2.6%) | 8 | 28532 (2.7%) | 8 | 29586 (2.8%) | 8 | 31645 (2.7%) | 8 | 32660 (2.8%) | 8 | 30893 (2.8%) | 8 | 34737 (2.8%) | 8 | 42793 (3.0%) | 7 | 50121 (3.0%) | 7 | 56182 (3.1%) | 7 |
| Systemic Lupus and Connective Tissue Disorders | 334480 (2.6%) | 8 | 35039 (3.3%) | 5 | 35778 (3.4%) | 5 | 35834 (3.3%) | 5 | 40069 (3.5%) | 6 | 41232 (3.5%) | 6 | 39503 (3.5%) | 6 | 37701 (3.0%) | 7 | 21349 (1.5%) | 12 | 23404 (1.4%) | 13 | 24571 (1.4%) | 13 |
| Trauma-Related Disorders | 291673 (2.3%) | 9 | 25242 (2.3%) | 9 | 25618 (2.4%) | 9 | 25937 (2.4%) | 9 | 28353 (2.5%) | 9 | 29219 (2.5%) | 9 | 27939 (2.5%) | 9 | 29882 (2.4%) | 9 | 30340 (2.1%) | 8 | 33366 (2.0%) | 9 | 35777 (2.0%) | 9 |
| Otitis Media and Related Conditions | 265143 (2.1%) | 10 | 25079 (2.3%) | 10 | 24927 (2.4%) | 10 | 24286 (2.3%) | 10 | 24406 (2.1%) | 10 | 24166 (2.0%) | 10 | 21486 (1.9%) | 10 | 24567 (2.0%) | 10 | 28538 (2.0%) | 9 | 32559 (1.9%) | 10 | 35129 (1.9%) | 10 |
| **Top 5 physician specialties, *n* (%)** | | | | | | | | | | | | | | | | | | | | | | |
| Family Practice | 3210015 (34.7%) | 1 | 292926 (33.3%) | 1 | 295417 (34.2%) | 1 | 293388 (34.2%) | 1 | 313730 (34.7%) | 1 | 310360 (34.5%) | 1 | 283435 (34.7%) | 1 | 304843 (34.8%) | 1 | 341731 (35.3%) | 1 | 383115 (35.6%) | 1 | 391070 (35.5%) | 1 |
| Internal Medicine | 1448382 (15.7%) | 2 | 131121 (14.9%) | 2 | 131498 (15.2%) | 2 | 129607 (15.1%) | 2 | 138626 (15.3%) | 2 | 139712 (15.5%) | 2 | 125164 (15.3%) | 2 | 137520 (15.7%) | 2 | 155342 (16.1%) | 2 | 177018 (16.5%) | 2 | 182774 (16.6%) | 2 |
| Emergency Medicine | 876695 (9.5%) | 3 | 73291 (8.3%) | 4 | 71201 (8.3%) | 4 | 75215 (8.8%) | 4 | 84157 (9.3%) | 3 | 85371 (9.5%) | 3 | 82584 (10.1%) | 3 | 88899 (10.2%) | 3 | 98999 (10.2%) | 3 | 109228 (10.2%) | 3 | 107750 (9.8%) | 3 |
| Pediatrics | 739557 (8.0%) | 4 | 103541 (11.8%) | 3 | 91011 (10.5%) | 3 | 86072 (10.0%) | 3 | 81859 (9.1%) | 4 | 73582 (8.2%) | 4 | 63798 (7.8%) | 4 | 60784 (6.9%) | 4 | 59613 (6.2%) | 4 | 61426 (5.7%) | 4 | 57871 (5.3%) | 4 |
| Surgery | 457297 (4.9%) | 5 | 40875 (4.6%) | 5 | 41391 (4.8%) | 5 | 41088 (4.8%) | 5 | 44761 (5.0%) | 5 | 46072 (5.1%) | 5 | 40420 (4.9%) | 5 | 44054 (5.0%) | 5 | 48525 (5.0%) | 5 | 53526 (5.0%) | 5 | 56585 (5.1%) | 5 |
| Note: |  |  |  |  |  |  |  |  |  |  |  |  |  |  |  |  |  |  |  |  |  |  |
| Abbreviation: **URI**: upper respiratory infection; **COPD**: chronic obstructive pulmonary disease. | | | | | | | | | | | | | | | | | | | | | | |

| **Table S5b**. Trend of top 10 indications and top 5 physician specialties of short-term oral corticosteroids use from 2009-2018 in Taiwan | | | | | | | | | | | | | | | | | | | | | | |
| --- | --- | --- | --- | --- | --- | --- | --- | --- | --- | --- | --- | --- | --- | --- | --- | --- | --- | --- | --- | --- | --- | --- |
|  | **Overall** | | **2009** | | **2010** | | **2011** | | **2012** | | **2013** | | **2014** | | **2015** | | **2016** | | **2017** | | **2018** | |
|  | ***n (%)*** | ***Rank*** | ***n (%)*** | ***Rank*** | ***n (%)*** | ***Rank*** | ***n (%)*** | ***Rank*** | ***n (%)*** | ***Rank*** | ***n (%)*** | ***Rank*** | ***n (%)*** | ***Rank*** | ***n (%)*** | ***Rank*** | ***n (%)*** | ***Rank*** | ***n (%)*** | ***Rank*** | ***n (%)*** | ***Rank*** |
| **Top 10 indications, *n* (%)** | | | | | | | | | | | | | | | | | | | | | | |
| Acute Bronchitis and URI | 17847113 (30.5%) | 1 | 1685520 (31.7%) | 1 | 1773439 (32.1%) | 1 | 2005031 (34.2%) | 1 | 1861410 (32.0%) | 1 | 1679861 (29.9%) | 1 | 1735074 (29.8%) | 1 | 1664174 (28.3%) | 1 | 1818374 (29.8%) | 1 | 1751412 (28.7%) | 1 | 1872540 (29.3%) | 1 |
| Allergic Reactions | 13299400 (22.8%) | 2 | 1270372 (23.9%) | 2 | 1320514 (23.9%) | 2 | 1328185 (22.6%) | 2 | 1345893 (23.2%) | 2 | 1379466 (24.6%) | 2 | 1435312 (24.7%) | 2 | 1502489 (25.5%) | 2 | 1238173 (20.3%) | 2 | 1235254 (20.2%) | 2 | 1242985 (19.5%) | 2 |
| Skin Disorders | 6711518 (11.5%) | 3 | 535586 (10.1%) | 4 | 535681 (9.7%) | 4 | 539263 (9.2%) | 4 | 558345 (9.6%) | 4 | 554914 (9.9%) | 4 | 573110 (9.9%) | 4 | 624486 (10.6%) | 3 | 917151 (15.0%) | 3 | 935650 (15.3%) | 3 | 934469 (14.6%) | 3 |
| COPD, Asthma, and Other Respiratory Conditions | 6224763 (10.6%) | 4 | 588472 (11.1%) | 3 | 614410 (11.1%) | 3 | 647962 (11.0%) | 3 | 640014 (11.0%) | 3 | 600543 (10.7%) | 3 | 618431 (10.6%) | 3 | 614168 (10.4%) | 4 | 615800 (10.1%) | 4 | 628271 (10.3%) | 4 | 654145 (10.2%) | 4 |
| Tonsillitis | 1844452 (3.2%) | 5 | 150100 (2.8%) | 5 | 148804 (2.7%) | 6 | 170764 (2.9%) | 5 | 176942 (3.0%) | 5 | 183835 (3.3%) | 5 | 195224 (3.4%) | 5 | 179871 (3.1%) | 6 | 199373 (3.3%) | 5 | 202333 (3.3%) | 5 | 237038 (3.7%) | 5 |
| Trauma-Related Disorders | 1727137 (3.0%) | 6 | 143026 (2.7%) | 6 | 160164 (2.9%) | 5 | 162996 (2.8%) | 6 | 171699 (3.0%) | 6 | 171846 (3.1%) | 6 | 179480 (3.1%) | 6 | 184247 (3.1%) | 5 | 171163 (2.8%) | 7 | 167210 (2.7%) | 7 | 189996 (3.0%) | 7 |
| Infectious Diseases | 1538664 (2.6%) | 7 | 132838 (2.5%) | 7 | 138811 (2.5%) | 7 | 138747 (2.4%) | 7 | 145158 (2.5%) | 7 | 149539 (2.7%) | 7 | 158511 (2.7%) | 7 | 165877 (2.8%) | 7 | 164817 (2.7%) | 8 | 165308 (2.7%) | 8 | 173552 (2.7%) | 8 |
| Osteoarthritis and Other Non-Traumatic Joint Disorders | 1292367 (2.2%) | 8 | 88490 (1.7%) | 9 | 88407 (1.6%) | 10 | 93435 (1.6%) | 9 | 105457 (1.8%) | 9 | 107602 (1.9%) | 9 | 107580 (1.8%) | 9 | 115084 (2.0%) | 9 | 186811 (3.1%) | 6 | 194002 (3.2%) | 6 | 198327 (3.1%) | 6 |
| Other CNS Disorders | 1082442 (1.9%) | 9 | 88630 (1.7%) | 8 | 92097 (1.7%) | 9 | 91984 (1.6%) | 10 | 102570 (1.8%) | 10 | 104233 (1.9%) | 10 | 107570 (1.8%) | 10 | 111922 (1.9%) | 10 | 117422 (1.9%) | 9 | 126811 (2.1%) | 9 | 132619 (2.1%) | 9 |
| Systemic Lupus and Connective Tissue Disorders | 740287 (1.3%) | 10 | 85726 (1.6%) | 11 | 93226 (1.7%) | 8 | 100949 (1.7%) | 8 | 113378 (2.0%) | 8 | 110872 (2.0%) | 8 | 116389 (2.0%) | 8 | 118847 (2.0%) | 8 | 28988 (0.5%) | 18 | 31248 (0.5%) | 18 | 31843 (0.5%) | 18 |
| **Top 5 physician specialties, *n* (%)** | | | | | | | | | | | | | | | | | | | | | | |
| Dermatology | 15057609 (25.8%) | 1 | 1251937 (23.5%) | 2 | 1313561 (23.8%) | 1 | 1353615 (23.1%) | 2 | 1394356 (24.0%) | 1 | 1440148 (25.7%) | 1 | 1517410 (26.1%) | 1 | 1644981 (28.0%) | 1 | 1683530 (27.6%) | 1 | 1712195 (28.1%) | 1 | 1745876 (27.3%) | 1 |
| Family Practice | 11901704 (20.4%) | 2 | 1275767 (24.0%) | 1 | 1296610 (23.5%) | 2 | 1360024 (23.2%) | 1 | 1282257 (22.1%) | 2 | 1155809 (20.6%) | 2 | 1141120 (19.6%) | 2 | 1109625 (18.9%) | 2 | 1097732 (18.0%) | 3 | 1077491 (17.7%) | 3 | 1105269 (17.3%) | 3 |
| Otolaryngology | 10700501 (18.3%) | 3 | 874893 (16.4%) | 3 | 935251 (16.9%) | 3 | 1003069 (17.1%) | 3 | 1023076 (17.6%) | 3 | 1023275 (18.2%) | 3 | 1073229 (18.5%) | 3 | 1080377 (18.4%) | 3 | 1177684 (19.3%) | 2 | 1195864 (19.6%) | 2 | 1313783 (20.6%) | 2 |
| Pediatrics | 7034318 (12.0%) | 4 | 647365 (12.2%) | 5 | 713831 (12.9%) | 4 | 770116 (13.1%) | 4 | 729807 (12.6%) | 4 | 651614 (11.6%) | 5 | 701214 (12.1%) | 4 | 667278 (11.3%) | 4 | 700930 (11.5%) | 4 | 709250 (11.6%) | 4 | 742913 (11.6%) | 4 |
| Internal Medicine | 6825969 (11.7%) | 5 | 701536 (13.2%) | 4 | 685444 (12.4%) | 5 | 754606 (12.9%) | 5 | 721565 (12.4%) | 5 | 675790 (12%) | 4 | 686321 (11.8%) | 5 | 656725 (11.2%) | 5 | 678453 (11.1%) | 5 | 631181 (10.3%) | 5 | 634348 (9.9%) | 5 |
| Note: | | | | | | | | | | | | | | | | | | | | | | |
| Abbreviation: **URI**: upper respiratory infection; **COPD**: chronic obstructive pulmonary disease; CNS: central nervous system. | | | | | | | | | | | | | | | | | | | | | | |
