# Supplementary material for: Prevalence and prescribing patterns of oral corticosteroids in the United States, Taiwan, and Denmark, 2009–2018
Source: Clin Transl Sci. 2023 Oct 6;16(12):2565–76. doi: 10.1111/cts.13649 (PMC10719491; doi:10.1111/cts.13649)
Supplement: Supplementary file 10 — Table S6 [file CTS-16-2565-s002.docx]

| **Table S6a.** Trend of top 10 indications and top 5 physician specialties of medium-term oral corticosteroids use from 2009-2018 in the USA | | | | | | | | | | | | | | | | | | | | | | |
| --- | --- | --- | --- | --- | --- | --- | --- | --- | --- | --- | --- | --- | --- | --- | --- | --- | --- | --- | --- | --- | --- | --- |
|  | **Overall** | | **2009** | | **2010** | | **2011** | | **2012** | | **2013** | | **2014** | | **2015** | | **2016** | | **2017** | | **2018** | |
|  | ***n (%)*** | ***Rank*** | ***n (%)*** | ***Rank*** | ***n (%)*** | ***Rank*** | ***n (%)*** | ***Rank*** | ***n (%)*** | ***Rank*** | ***n (%)*** | ***Rank*** | ***n (%)*** | ***Rank*** | ***n (%)*** | ***Rank*** | ***n (%)*** | ***Rank*** | ***n (%)*** | ***Rank*** | ***n (%)*** | ***Rank*** |
| **Top 10 indications, *n* (%)** | | | | | | | | | | | | | | | | | | | | | | |
| COPD, Asthma, and Other Respiratory Conditions | 308377 (20.3%) | 1 | 26317 (20.7%) | 1 | 24264 (20.3%) | 1 | 25959 (20.0%) | 1 | 30051 (20.6%) | 1 | 28836 (20.7%) | 1 | 25105 (20.3%) | 1 | 29624 (20.6%) | 1 | 32332 (19.6%) | 1 | 40614 (20.5%) | 1 | 45275 (20.2%) | 1 |
| Acute Bronchitis and URI | 159544 (10.5%) | 2 | 13840 (10.9%) | 2 | 13306 (11.1%) | 2 | 14859 (11.5%) | 2 | 17788 (12.2%) | 2 | 15223 (10.9%) | 2 | 11867 (9.6%) | 2 | 13828 (9.6%) | 2 | 17255 (10.5%) | 2 | 20667 (10.4%) | 2 | 20911 (9.3%) | 2 |
| Osteoarthritis and Other Non-Traumatic Joint Disorders | 120732 (8.0%) | 3 | 8558 (6.7%) | 3 | 8173 (6.8%) | 3 | 9240 (7.1%) | 3 | 10081 (6.9%) | 3 | 10150 (7.3%) | 3 | 9304 (7.5%) | 3 | 11447 (8.0%) | 3 | 15261 (9.2%) | 3 | 18113 (9.1%) | 3 | 20405 (9.1%) | 3 |
| Back Problems | 75706 (5.0%) | 4 | 5215 (4.1%) | 5 | 5337 (4.5%) | 5 | 6058 (4.7%) | 5 | 7214 (5.0%) | 5 | 6691 (4.8%) | 5 | 6448 (5.2%) | 4 | 7693 (5.4%) | 4 | 8722 (5.3%) | 4 | 10916 (5.5%) | 4 | 11412 (5.1%) | 5 |
| Allergic Reactions | 75700 (5.0%) | 5 | 7478 (5.9%) | 4 | 7349 (6.1%) | 4 | 7656 (5.9%) | 4 | 8321 (5.7%) | 4 | 7539 (5.4%) | 4 | 6429 (5.2%) | 5 | 7264 (5.1%) | 5 | 7179 (4.3%) | 7 | 7904 (4.0%) | 7 | 8581 (3.8%) | 7 |
| Cancer | 67396 (4.4%) | 6 | 4905 (3.9%) | 7 | 4554 (3.8%) | 6 | 4659 (3.6%) | 7 | 5395 (3.7%) | 6 | 5528 (4.0%) | 6 | 5357 (4.3%) | 6 | 5983 (4.2%) | 6 | 7338 (4.4%) | 6 | 9410 (4.7%) | 5 | 14267 (6.4%) | 4 |
| Skin Disorders | 61698 (4.1%) | 7 | 4324 (3.4%) | 9 | 4358 (3.6%) | 8 | 4608 (3.6%) | 8 | 5286 (3.6%) | 8 | 5026 (3.6%) | 8 | 4830 (3.9%) | 8 | 5424 (3.8%) | 7 | 8135 (4.9%) | 5 | 9266 (4.7%) | 6 | 10441 (4.7%) | 6 |
| Systemic Lupus and Connective Tissue Disorders | 48127 (3.2%) | 8 | 4927 (3.9%) | 6 | 4508 (3.8%) | 7 | 4996 (3.9%) | 6 | 5380 (3.7%) | 7 | 5367 (3.9%) | 7 | 4884 (3.9%) | 7 | 4978 (3.5%) | 8 | 3931 (2.4%) | 11 | 4312 (2.2%) | 12 | 4844 (2.2%) | 13 |
| Other Stomach and Intestinal Disorders | 45298 (3.0%) | 9 | 4345 (3.4%) | 8 | 3851 (3.2%) | 9 | 4125 (3.2%) | 9 | 4243 (2.9%) | 9 | 4217 (3.0%) | 9 | 3857 (3.1%) | 9 | 4092 (2.8%) | 10 | 5108 (3.1%) | 8 | 5481 (2.8%) | 10 | 5979 (2.7%) | 10 |
| Other Endocrine, Nutritional & Immune Disorder | 42846 (2.8%) | 10 | 3059 (2.4%) | 11 | 2990 (2.5%) | 11 | 3964 (3.1%) | 10 | 3952 (2.7%) | 10 | 3949 (2.8%) | 10 | 3726 (3.0%) | 10 | 4167 (2.9%) | 9 | 4652 (2.8%) | 10 | 5798 (2.9%) | 9 | 6589 (2.9%) | 9 |
| **Top 5 physician specialties, *n* (%)** | | | | | | | | | | | | | | | | | | | | | | |
| Family Practice | 285382 (23.8%) | 1 | 24404 (22.7%) | 1 | 23530 (23.3%) | 1 | 25647 (23.4%) | 1 | 28512 (23.5%) | 1 | 26687 (23.3%) | 1 | 23825 (23.9%) | 1 | 27513 (24.2%) | 1 | 30888 (24.2%) | 1 | 36547 (24.9%) | 1 | 37829 (24.0%) | 1 |
| Internal Medicine | 207203 (17.3%) | 2 | 16721 (15.6%) | 2 | 16424 (16.2%) | 2 | 18275 (16.7%) | 2 | 19802 (16.3%) | 2 | 19250 (16.8%) | 2 | 16737 (16.8%) | 2 | 20377 (18.0%) | 2 | 23244 (18.2%) | 2 | 27139 (18.5%) | 2 | 29234 (18.5%) | 2 |
| Rheumatology | 102882 (8.6%) | 3 | 9617 (9.0%) | 3 | 8691 (8.6%) | 3 | 9346 (8.5%) | 3 | 9396 (7.8%) | 3 | 10175 (8.9%) | 3 | 9199 (9.2%) | 3 | 9932 (8.8%) | 3 | 11183 (8.8%) | 3 | 12304 (8.4%) | 3 | 13039 (8.3%) | 3 |
| Pulmonary Disease | 69002 (5.8%) | 4 | 5961 (5.6%) | 4 | 5428 (5.4%) | 5 | 5572 (5.1%) | 4 | 6247 (5.2%) | 5 | 6379 (5.6%) | 4 | 5789 (5.8%) | 4 | 6602 (5.8%) | 4 | 7685 (6.0%) | 4 | 9132 (6.2%) | 4 | 10207 (6.5%) | 4 |
| Otolaryngology | 57088 (4.8%) | 5 | 5747 (5.4%) | 5 | 5589 (5.5%) | 4 | 5470 (5.0%) | 5 | 6047 (5.0%) | 6 | 5601 (4.9%) | 5 | 4728 (4.7%) | 5 | 5286 (4.7%) | 6 | 5881 (4.6%) | 6 | 6282 (4.3%) | 6 | 6457 (4.1%) | 7 |
| Note: |  |  |  |  |  |  |  |  |  |  |  |  |  |  |  |  |  |  |  |  |  |  |
| Abbreviation: **COPD**: chronic obstructive pulmonary disease; **URI**: upper respiratory infection. | | | | | | | | | | | | | | | | | | | | | | |

| **Table S6b.** Trend of top 10 indications and top 5 physician specialties of medium-term oral corticosteroids use from 2009-2018 in Taiwan | | | | | | | | | | | | | | | | | | | | | | |
| --- | --- | --- | --- | --- | --- | --- | --- | --- | --- | --- | --- | --- | --- | --- | --- | --- | --- | --- | --- | --- | --- | --- |
|  | **Overall** | | **2009** | | **2010** | | **2011** | | **2012** | | **2013** | | **2014** | | **2015** | | **2016** | | **2017** | | **2018** | |
|  | ***n (%)*** | ***Rank*** | ***n (%)*** | ***Rank*** | ***n (%)*** | ***Rank*** | ***n (%)*** | ***Rank*** | ***n (%)*** | ***Rank*** | ***n (%)*** | ***Rank*** | ***n (%)*** | ***Rank*** | ***n (%)*** | ***Rank*** | ***n (%)*** | ***Rank*** | ***n (%)*** | ***Rank*** | ***n (%)*** | ***Rank*** |
| **Top 10 indications, *n* (%)** | | | | | | | | | | | | | | | | | | | | | | |
| Acute Bronchitis and URI | 2987424 (20.2%) | 1 | 276381 (23.6%) | 1 | 296448 (23.8%) | 1 | 320710 (23.9%) | 1 | 321207 (22.2%) | 1 | 271110 (19.4%) | 2 | 287362 (19.4%) | 2 | 272680 (17.6%) | 3 | 307260 (18.9%) | 1 | 302043 (17.7%) | 2 | 331996 (18.1%) | 2 |
| COPD, Asthma, and Other Respiratory Conditions | 2742169 (18.6%) | 2 | 223660 (19.1%) | 2 | 232591 (18.7%) | 2 | 243285 (18.2%) | 2 | 270160 (18.7%) | 2 | 266121 (19.0%) | 3 | 275205 (18.6%) | 3 | 287692 (18.6%) | 2 | 296671 (18.3%) | 2 | 309881 (18.2%) | 1 | 334458 (18.2%) | 1 |
| Allergic Reactions | 2668185 (18.0%) | 3 | 207014 (17.7%) | 3 | 221266 (17.8%) | 3 | 235019 (17.5%) | 3 | 259211 (18.0%) | 3 | 272728 (19.5%) | 1 | 288329 (19.5%) | 1 | 318469 (20.6%) | 1 | 273780 (16.9%) | 3 | 290863 (17.1%) | 3 | 300858 (16.4%) | 3 |
| Skin Disorders | 1515846 (10.3%) | 4 | 103668 (8.9%) | 4 | 103739 (8.3%) | 4 | 110032 (8.2%) | 4 | 123851 (8.6%) | 4 | 125049 (8.9%) | 4 | 130976 (8.9%) | 4 | 151448 (9.8%) | 4 | 207585 (12.8%) | 4 | 224220 (13.2%) | 4 | 232067 (12.7%) | 4 |
| Osteoarthritis and Other Non-Traumatic Joint Disorders | 829661 (5.6%) | 5 | 56332 (4.8%) | 5 | 59150 (4.8%) | 5 | 63805 (4.8%) | 5 | 72709 (5.0%) | 5 | 72531 (5.2%) | 5 | 77009 (5.2%) | 5 | 81709 (5.3%) | 5 | 109848 (6.8%) | 5 | 113358 (6.7%) | 5 | 116552 (6.4%) | 5 |
| Other Endocrine, Nutritional & Immune Disorders | 407076 (2.8%) | 6 | 26243 (2.2%) | 8 | 29139 (2.3%) | 8 | 34000 (2.5%) | 8 | 36748 (2.5%) | 8 | 37443 (2.7%) | 8 | 41376 (2.8%) | 7 | 43117 (2.8%) | 7 | 47916 (2.9%) | 6 | 52296 (3.1%) | 6 | 54809 (3.0%) | 6 |
| Systemic Lupus and Connective Tissue Disorders | 293381 (2.0%) | 7 | 29888 (2.6%) | 7 | 36053 (2.9%) | 6 | 39325 (2.9%) | 7 | 43463 (3.0%) | 6 | 43566 (3.1%) | 6 | 47838 (3.2%) | 6 | 52084 (3.4%) | 6 | 21540 (1.3%) | 14 | 23335 (1.4%) | 14 | 24177 (1.3%) | 14 |
| Back Problems | 272395 (1.8%) | 8 | 33739 (2.9%) | 6 | 35609 (2.9%) | 7 | 39920 (3.0%) | 6 | 41748 (2.9%) | 7 | 38959 (2.8%) | 7 | 40303 (2.7%) | 8 | 41404 (2.7%) | 8 | 36642 (2.3%) | 7 | 39059 (2.3%) | 7 | 42734 (2.3%) | 7 |
| Tonsillitis | 222289 (1.5%) | 9 | 16518 (1.4%) | 12 | 17448 (1.4%) | 12 | 19907 (1.5%) | 11 | 21601 (1.5%) | 13 | 18073 (1.3%) | 13 | 22151 (1.5%) | 13 | 20457 (1.3%) | 14 | 25673 (1.6%) | 11 | 28172 (1.7%) | 12 | 31935 (1.7%) | 12 |
| Trauma-Related Disorders | 213408 (1.4%) | 10 | 23021 (2.0%) | 10 | 25672 (2.1%) | 9 | 28531 (2.1%) | 9 | 31239 (2.2%) | 9 | 31152 (2.2%) | 9 | 31312 (2.1%) | 9 | 31989 (2.1%) | 9 | 29754 (1.8%) | 10 | 30644 (1.8%) | 10 | 35215 (1.9%) | 10 |
| **Top 5 physician specialties, *n* (%)** | | | | | | | | | | | | | | | | | | | | | | |
| Family Practice | 3336857 (22.6%) | 1 | 312997 (26.8%) | 1 | 332027 (26.7%) | 1 | 348440 (26.0%) | 1 | 362663 (25.1%) | 1 | 321983 (23.0%) | 1 | 326526 (22.1%) | 1 | 326873 (21.1%) | 2 | 318465 (19.6%) | 2 | 335064 (19.7%) | 2 | 351819 (19.2%) | 2 |
| Dermatology | 2872001 (19.4%) | 2 | 193114 (16.5%) | 2 | 203568 (16.4%) | 2 | 224669 (16.8%) | 2 | 253596 (17.6%) | 2 | 269281 (19.3%) | 2 | 291503 (19.7%) | 2 | 336010 (21.7%) | 1 | 343215 (21.1%) | 1 | 369335 (21.7%) | 1 | 387710 (21.2%) | 1 |
| Internal Medicine | 2001072 (13.5%) | 3 | 176082 (15.1%) | 3 | 178021 (14.3%) | 4 | 196472 (14.7%) | 3 | 206963 (14.3%) | 3 | 197908 (14.2%) | 3 | 205822 (13.9%) | 3 | 202224 (13.1%) | 3 | 212355 (13.1%) | 3 | 207425 (12.2%) | 4 | 217800 (11.9%) | 4 |
| Pediatrics | 1869723 (12.7%) | 4 | 165981 (14.2%) | 4 | 185185 (14.9%) | 3 | 187533 (14.0%) | 4 | 191285 (13.3%) | 4 | 170467 (12.2%) | 4 | 186759 (12.6%) | 4 | 182601 (11.8%) | 4 | 193798 (11.9%) | 5 | 197947 (11.6%) | 5 | 208167 (11.4%) | 5 |
| Otolaryngology | 1675793 (11.3%) | 5 | 117026 (10.0%) | 5 | 128964 (10.4%) | 5 | 135045 (10.1%) | 5 | 152328 (10.6%) | 5 | 148478 (10.6%) | 5 | 160881 (10.9%) | 5 | 175830 (11.4%) | 5 | 200575 (12.4%) | 4 | 213208 (12.5%) | 3 | 243458 (13.3%) | 3 |
| Note: |  |  |  |  |  |  |  |  |  |  |  |  |  |  |  |  |  |  |  |  |  |  |
| Abbreviation: **COPD**: chronic obstructive pulmonary disease; **URI**: upper respiratory infection. | | | | | | | | | | | | | | | | | | | | | | |
