# Supplementary material for: Prevalence and prescribing patterns of oral corticosteroids in the United States, Taiwan, and Denmark, 2009–2018
Source: Clin Transl Sci. 2023 Oct 6;16(12):2565–76. doi: 10.1111/cts.13649 (PMC10719491; doi:10.1111/cts.13649)
Supplement: Supplementary file 11 — Table S7 [file CTS-16-2565-s004.docx]

| **Table S7a.** Trend of top 10 indications and top 5 physician specialties of long-term oral corticosteroids use from 2009-2018 in the USA | | | | | | | | | | | | | | | | | | | | | | |
| --- | --- | --- | --- | --- | --- | --- | --- | --- | --- | --- | --- | --- | --- | --- | --- | --- | --- | --- | --- | --- | --- | --- |
|  | **Overall** | | **2009** | | **2010** | | **2011** | | **2012** | | **2013** | | **2014** | | **2015** | | **2016** | | **2017** | | **2018** | |
|  | ***n (%)*** | ***Rank*** | ***n (%)*** | ***Rank*** | ***n (%)*** | ***Rank*** | ***n (%)*** | ***Rank*** | ***n (%)*** | ***Rank*** | ***n (%)*** | ***Rank*** | ***n (%)*** | ***Rank*** | ***n (%)*** | ***Rank*** | ***n (%)*** | ***Rank*** | ***n (%)*** | ***Rank*** | ***n (%)*** | ***Rank*** |
| **Top 10 indications, *n* (%)** | | | | | | | | | | | | | | | | | | | | | | |
| Osteoarthritis and Other Non-Traumatic Joint Disorders | 191990 (14.8%) | 1 | 17762 (14.9%) | 1 | 15417 (15.3%) | 1 | 15735 (15.3%) | 1 | 17590 (15.4%) | 1 | 17461 (15.3%) | 1 | 16276 (15.4%) | 1 | 17732 (14.6%) | 1 | 21711 (15.3%) | 1 | 24489 (14.5%) | 1 | 27817 (13.5%) | 1 |
| COPD, Asthma, and Other Respiratory Conditions | 133349 (10.3%) | 2 | 9696 (8.1%) | 3 | 8711 (8.6%) | 3 | 9441 (9.2%) | 3 | 10493 (9.2%) | 3 | 10880 (9.5%) | 3 | 10295 (9.8%) | 3 | 12683 (10.5%) | 2 | 15296 (10.8%) | 2 | 20440 (12.1%) | 2 | 25414 (12.4%) | 2 |
| Systemic Lupus and Connective Tissue Disorders | 124137 (9.6%) | 3 | 11131 (9.4%) | 2 | 9759 (9.7%) | 2 | 10418 (10.2%) | 2 | 12032 (10.5%) | 2 | 11806 (10.3%) | 2 | 10732 (10.2%) | 2 | 11935 (9.9%) | 3 | 13656 (9.6%) | 3 | 15462 (9.1%) | 3 | 17206 (8.4%) | 3 |
| Cancer | 55807 (4.3%) | 4 | 2913 (2.4%) | 8 | 2723 (2.7%) | 8 | 3131 (3.1%) | 7 | 3449 (3.0%) | 7 | 3906 (3.4%) | 5 | 3859 (3.7%) | 4 | 4719 (3.9%) | 4 | 6215 (4.4%) | 4 | 9207 (5.4%) | 4 | 15685 (7.6%) | 4 |
| Other Endocrine, Nutritional & Immune Disorders | 43563 (3.4%) | 5 | 4000 (3.4%) | 5 | 3398 (3.4%) | 5 | 3782 (3.7%) | 4 | 3835 (3.4%) | 5 | 4024 (3.5%) | 4 | 3479 (3.3%) | 5 | 3687 (3.0%) | 6 | 4958 (3.5%) | 6 | 5611 (3.3%) | 6 | 6789 (3.3%) | 6 |
| Skin Disorders | 42950 (3.3%) | 6 | 3488 (2.9%) | 6 | 3257 (3.2%) | 6 | 3198 (3.1%) | 6 | 3531 (3.1%) | 6 | 3544 (3.1%) | 7 | 3328 (3.2%) | 6 | 3845 (3.2%) | 5 | 5135 (3.6%) | 5 | 6229 (3.7%) | 5 | 7395 (3.6%) | 5 |
| Other Stomach and Intestinal Disorders | 39804 (3.1%) | 7 | 4164 (3.5%) | 4 | 3650 (3.6%) | 4 | 3501 (3.4%) | 5 | 3840 (3.4%) | 4 | 3570 (3.1%) | 6 | 3218 (3.0%) | 7 | 3544 (2.9%) | 7 | 4187 (3.0%) | 8 | 4703 (2.8%) | 9 | 5427 (2.6%) | 9 |
| Hypertension | 34043 (2.6%) | 8 | 2872 (2.4%) | 9 | 2491 (2.5%) | 9 | 2557 (2.5%) | 9 | 2826 (2.5%) | 10 | 2873 (2.5%) | 10 | 2708 (2.6%) | 9 | 3215 (2.7%) | 9 | 3890 (2.7%) | 9 | 4859 (2.9%) | 8 | 5752 (2.8%) | 8 |
| Nervous System Disorders | 33932 (2.6%) | 9 | 2441 (2.1%) | 12 | 2092 (2.1%) | 12 | 2183 (2.1%) | 12 | 2516 (2.2%) | 11 | 2746 (2.4%) | 11 | 2681 (2.5%) | 10 | 3161 (2.6%) | 10 | 4404 (3.1%) | 7 | 5199 (3.1%) | 7 | 6509 (3.2%) | 7 |
| Acute Bronchitis and URI | 32764 (2.5%) | 10 | 3338 (2.8%) | 7 | 2956 (2.9%) | 7 | 2851 (2.8%) | 8 | 3010 (2.6%) | 8 | 2928 (2.6%) | 8 | 2391 (2.3%) | 12 | 2856 (2.4%) | 11 | 3536 (2.5%) | 11 | 4231 (2.5%) | 11 | 4667 (2.3%) | 13 |
| **Top 5 physician specialties, *n* (%)** | | | | | | | | | | | | | | | | | | | | | | |
| Rheumatology | 344334 (31.6%) | 1 | 34245 (33.1%) | 1 | 29347 (33.2%) | 1 | 29337 (32.8%) | 1 | 32803 (33.0%) | 1 | 32768 (32.9%) | 1 | 30061 (33.1%) | 1 | 33479 (32.5%) | 1 | 37302 (31.4%) | 1 | 40538 (29.4%) | 1 | 44454 (27.9%) | 1 |
| Internal Medicine | 163919 (15.0%) | 2 | 13493 (13.0%) | 2 | 11883 (13.4%) | 2 | 12996 (14.5%) | 2 | 14612 (14.7%) | 2 | 14372 (14.4%) | 2 | 13656 (15.0%) | 2 | 15765 (15.3%) | 2 | 18745 (15.8%) | 2 | 22616 (16.4%) | 2 | 25781 (16.2%) | 2 |
| Family Practice | 150830 (13.8%) | 3 | 12773 (12.3%) | 3 | 11016 (12.5%) | 3 | 11492 (12.8%) | 3 | 12938 (13.0%) | 3 | 12964 (13.0%) | 3 | 12768 (14.0%) | 3 | 14697 (14.3%) | 3 | 17446 (14.7%) | 3 | 20946 (15.2%) | 3 | 23790 (14.9%) | 3 |
| Pulmonology | 63271 (5.8%) | 4 | 5148 (5.0%) | 5 | 4682 (5.3%) | 4 | 4702 (5.3%) | 4 | 5313 (5.3%) | 4 | 5484 (5.5%) | 4 | 4688 (5.2%) | 4 | 6002 (5.8%) | 4 | 7401 (6.2%) | 4 | 9017 (6.5%) | 4 | 10834 (6.8%) | 4 |
| Nephrology | 40236 (3.7%) | 5 | 5374 (5.2%) | 4 | 4011 (4.5%) | 5 | 3697 (4.1%) | 5 | 4018 (4.0%) | 5 | 3811 (3.8%) | 5 | 3315 (3.6%) | 5 | 3464 (3.4%) | 5 | 3833 (3.2%) | 6 | 4176 (3.0%) | 6 | 4537 (2.8%) | 6 |
| Note: |  |  |  |  |  |  |  |  |  |  |  |  |  |  |  |  |  |  |  |  |  |  |
| Abbreviation: **COPD**: chronic obstructive pulmonary disease; **URI**: upper respiratory infection. | | | | | | | | | | | | | | | | | | | | | | |

| **Table S7b**. Trend of top 10 indications and top 5 physician specialties of long-term oral corticosteroids use from 2009-2018 in Taiwan | | | | | | | | | | | | | | | | | | | | | | |
| --- | --- | --- | --- | --- | --- | --- | --- | --- | --- | --- | --- | --- | --- | --- | --- | --- | --- | --- | --- | --- | --- | --- |
|  | **Overall** | | **2009** | | **2010** | | **2011** | | **2012** | | **2013** | | **2014** | | **2015** | | **2016** | | **2017** | | **2018** | |
|  | ***n (%)*** | ***Rank*** | ***n (%)*** | ***Rank*** | ***n (%)*** | ***Rank*** | ***n (%)*** | ***Rank*** | ***n (%)*** | ***Rank*** | ***n (%)*** | ***Rank*** | ***n (%)*** | ***Rank*** | ***n (%)*** | ***Rank*** | ***n (%)*** | ***Rank*** | ***n (%)*** | ***Rank*** | ***n (%)*** | ***Rank*** |
| **Top 10 indications, *n* (%)** | | | | | | | | | | | | | | | | | | | | | | |
| Osteoarthritis and Other Non-Traumatic Joint Disorders | 2296452 (18.5%) | 1 | 174293 (20.4%) | 1 | 184970 (20.2%) | 1 | 200061 (19.7%) | 1 | 219421 (19.5%) | 1 | 228531 (19.2%) | 1 | 233777 (18.6%) | 1 | 242074 (18.1%) | 1 | 257087 (17.9%) | 1 | 268458 (17.1%) | 1 | 281127 (16.3%) | 1 |
| Systemic Lupus and Connective Tissues Disorders | 1711176 (13.8%) | 2 | 136233 (16.0%) | 2 | 146650 (16.0%) | 2 | 154880 (15.3%) | 2 | 166130 (14.7%) | 2 | 169631 (14.3%) | 2 | 175858 (14.0%) | 2 | 183848 (13.7%) | 2 | 182843 (12.7%) | 3 | 194006 (12.4%) | 3 | 199364 (11.6%) | 3 |
| COPD, Asthma, and Other Respiratory Conditions | 1668440 (13.4%) | 3 | 118508 (13.9%) | 3 | 120806 (13.1%) | 3 | 133815 (13.2%) | 3 | 156026 (13.9%) | 3 | 162637 (13.7%) | 3 | 174299 (13.9%) | 3 | 182503 (13.6%) | 3 | 189841 (13.2%) | 2 | 202509 (12.9%) | 2 | 225122 (13.0%) | 2 |
| Allergic Reactions | 809237 (6.5%) | 4 | 50908 (6.0%) | 5 | 54305 (5.9%) | 5 | 60911 (6.0%) | 6 | 69612 (6.2%) | 4 | 82673 (7.0%) | 4 | 88613 (7.1%) | 4 | 98588 (7.4%) | 4 | 90021 (6.3%) | 4 | 102021 (6.5%) | 5 | 110948 (6.4%) | 5 |
| Kidney Diseases | 670633 (5.4%) | 5 | 53365 (6.3%) | 4 | 57307 (6.2%) | 4 | 61123 (6.0%) | 5 | 65965 (5.9%) | 6 | 71880 (6.0%) | 5 | 78566 (6.3%) | 5 | 86514 (6.5%) | 5 | 59141 (4.1%) | 8 | 64829 (4.1%) | 8 | 70783 (4.1%) | 9 |
| Other endocrine, nutritional & immune disorder | 652537 (5.3%) | 6 | 42726 (5.0%) | 7 | 48032 (5.2%) | 7 | 53222 (5.3%) | 7 | 59925 (5.3%) | 7 | 64963 (5.5%) | 6 | 68256 (5.4%) | 6 | 72218 (5.4%) | 6 | 73463 (5.1%) | 7 | 79971 (5.1%) | 6 | 85648 (5.0%) | 8 |
| Acute Bronchitis and URI | 643475 (5.2%) | 7 | 47055 (5.5%) | 6 | 52861 (5.8%) | 6 | 62491 (6.2%) | 4 | 66440 (5.9%) | 5 | 59566 (5.0%) | 7 | 61867 (4.9%) | 7 | 59278 (4.4%) | 8 | 73876 (5.1%) | 6 | 73765 (4.7%) | 7 | 85922 (5.0%) | 7 |
| Skin disorders | 641823 (5.2%) | 8 | 34150 (4.0%) | 8 | 36322 (4.0%) | 8 | 39912 (3.9%) | 8 | 45505 (4.0%) | 8 | 52254 (4.4%) | 8 | 55292 (4.4%) | 8 | 65166 (4.9%) | 7 | 87009 (6.0%) | 5 | 104379 (6.7%) | 4 | 118469 (6.9%) | 4 |
| Cancer | 351284 (2.8%) | 9 | 11920 (1.4%) | 13 | 13156 (1.4%) | 14 | 15932 (1.6%) | 13 | 19025 (1.7%) | 13 | 23521 (2.0%) | 10 | 27991 (2.2%) | 10 | 34777 (2.6%) | 9 | 44414 (3.1%) | 10 | 60355 (3.9%) | 9 | 89517 (5.2%) | 6 |
| Hypertension | 280724 (2.3%) | 10 | 18303 (2.1%) | 9 | 20672 (2.2%) | 9 | 23109 (2.3%) | 9 | 26413 (2.3%) | 9 | 27349 (2.3%) | 9 | 29012 (2.3%) | 9 | 30073 (2.2%) | 10 | 34769 (2.4%) | 12 | 33803 (2.2%) | 12 | 36721 (2.1%) | 12 |
| **Top 5 physician specialties, *n* (%)** | | | | | | | | | | | | | | | | | | | | | | |
| Rheumatology | 4024129 (32.4%) | 1 | 292004 (34.2%) | 1 | 320207 (34.8%) | 1 | 348901 (34.4%) | 1 | 378237 (33.6%) | 1 | 403322 (33.9%) | 1 | 417439 (33.2%) | 1 | 435402 (32.5%) | 1 | 451016 (31.3%) | 1 | 478821 (30.6%) | 1 | 498780 (28.9%) | 1 |
| Family practice | 1329867 (10.7%) | 2 | 99869 (11.7%) | 2 | 109666 (11.9%) | 2 | 118003 (11.6%) | 2 | 134957 (12.0%) | 2 | 130155 (11.0%) | 2 | 129847 (10.3%) | 3 | 133567 (10.0%) | 2 | 138435 (9.6%) | 3 | 157279 (10.1%) | 2 | 178089 (10.3%) | 2 |
| Internal medicine | 1262202 (10.2%) | 3 | 98192 (11.5%) | 3 | 99035 (10.8%) | 3 | 111072 (11.0%) | 3 | 123887 (11.0%) | 3 | 126074 (10.6%) | 3 | 132475 (10.6%) | 2 | 133103 (1.0%0) | 3 | 139114 (9.7%) | 2 | 141830 (9.1%) | 3 | 157420 (9.1%) | 3 |
| Dermatology | 901965 (7.3%) | 4 | 50145 (5.9%) | 5 | 52359 (5.7%) | 5 | 59185 (5.8%) | 5 | 68248 (6.1%) | 5 | 80431 (6.8%) | 4 | 88383 (7.0%) | 4 | 104977 (7.8%) | 4 | 114970 (8.0%) | 4 | 133407 (8.5%) | 4 | 149860 (8.7%) | 4 |
| Pulmonology | 835064 (6.7%) | 5 | 48504 (5.7%) | 6 | 50702 (5.5%) | 6 | 59179 (5.8%) | 6 | 69443 (6.2%) | 4 | 75266 (6.3%) | 5 | 85168 (6.8%) | 5 | 92022 (6.9%) | 5 | 103427 (7.2%) | 5 | 116103 (7.4%) | 5 | 135250 (7.8%) | 5 |
| Note: |  |  |  |  |  |  |  |  |  |  |  |  |  |  |  |  |  |  |  |  |  |  |
| Abbreviation: **COPD**: chronic obstructive pulmonary disease; **URI**: upper respiratory infection. | | | | | | | | | | | | | | | | | | | | | | |
