# Supplementary material for: Prevalence and prescribing patterns of oral corticosteroids in the United States, Taiwan, and Denmark, 2009–2018
Source: Clin Transl Sci. 2023 Oct 6;16(12):2565–76. doi: 10.1111/cts.13649 (PMC10719491; doi:10.1111/cts.13649)
Supplement: Supplementary file 12 — Table S8 [file CTS-16-2565-s012.docx]

| **Table S8.** Trend of top 10 provider specialties of overall, short-, medium- and long-term oral corticosteroids use from 2009-2018 in the USA, including mid-level providers | | | | | | | | | | | | | | | | | | | | | | |
| --- | --- | --- | --- | --- | --- | --- | --- | --- | --- | --- | --- | --- | --- | --- | --- | --- | --- | --- | --- | --- | --- | --- |
|  | **Overall** | | **2009** | | **2010** | | **2011** | | **2012** | | **2013** | | **2014** | | **2015** | | **2016** | | **2017** | | **2018** | |
|  | ***Count (%)*** | ***Rank*** | ***Count (%)*** | ***Rank*** | ***Count (%)*** | ***Rank*** | ***Count (%)*** | ***Rank*** | ***Count (%)*** | ***Rank*** | ***Count (%)*** | ***Rank*** | ***Count (%)*** | ***Rank*** | ***Count (%)*** | ***Rank*** | ***Count (%)*** | ***Rank*** | ***Count (%)*** | ***Rank*** | ***Count (%)*** | ***Rank*** |
| **Overall oral corticosteroids use** | | | | | | | | | | | | | | | | | | | | | | |
| **Physician specialties, *n* (%)** | | | | | | | | | | | | | | | | | | | | | | |
| Family Practice | 3646227 (23.3%) | 1 | 330103 (25.0%) | 1 | 329963 (25.8%) | 1 | 330527 (25.3%) | 1 | 355180 (25.2%) | 1 | 350011 (24.4%) | 1 | 320028 (23.8%) | 1 | 347053 (23.0%) | 1 | 390065 (22.4%) | 1 | 440608 (21.6%) | 1 | 452689 (20.1%) | 1 |
| Internal Medicine | 1819504 (11.6%) | 2 | 161335 (12.2%) | 2 | 159805 (12.5%) | 2 | 160878 (12.3%) | 2 | 173040 (12.3%) | 2 | 173334 (12.1%) | 2 | 155557 (11.5%) | 2 | 173662 (11.5%) | 2 | 197331 (11.3%) | 3 | 226773 (11.1%) | 3 | 237789 (10.6%) | 3 |
| Nurse Practitioner | 1411067 (9.0%) | 3 | 41580 (3.1%) | 8 | 50723 (4.0%) | 5 | 64588 (4.9%) | 5 | 86141 (6.1%) | 5 | 104618 (7.3%) | 3 | 120756 (9.0%) | 3 | 156715 (10.4%) | 3 | 208481 (12.0%) | 2 | 264909 (13.0%) | 2 | 312556 (13.9%) | 2 |
| Physician Assistant | 1092711 (7.0%) | 4 | 38145 (2.9%) | 9 | 47000 (3.7%) | 7 | 59139 (4.5%) | 6 | 76527 (5.4%) | 6 | 90134 (6.3%) | 5 | 101114 (7.5%) | 4 | 127809 (8.5%) | 4 | 154919 (8.9%) | 4 | 187191 (9.2%) | 4 | 210733 (9.4%) | 4 |
| Emergency Medicine | 949565 (6.1%) | 5 | 78631 (6.0%) | 4 | 76234 (6.0%) | 4 | 80952 (6.2%) | 4 | 91526 (6.5%) | 3 | 92251 (6.4%) | 4 | 88580 (6.6%) | 5 | 96268 (6.4%) | 5 | 107402 (6.2%) | 5 | 119417 (5.9%) | 5 | 118304 (5.3%) | 5 |
| Pediatrics | 773048 (4.9%) | 6 | 107712 (8.2%) | 3 | 94379 (7.4%) | 3 | 90920 (7.0%) | 3 | 88638 (6.3%) | 4 | 77877 (5.4%) | 6 | 66051 (4.9%) | 6 | 62914 (4.2%) | 6 | 61555 (3.5%) | 6 | 63374 (3.1%) | 6 | 59628 (2.7%) | 8 |
| Rheumatology | 529352 (3.4%) | 7 | 50824 (3.8%) | 5 | 44956 (3.5%) | 9 | 45800 (3.5%) | 9 | 49807 (3.5%) | 8 | 51060 (3.6%) | 7 | 46628 (3.5%) | 7 | 51456 (3.4%) | 7 | 57611 (3.3%) | 7 | 62771 (3.1%) | 7 | 68439 (3.0%) | 6 |
| Surgery | 509471 (3.3%) | 8 | 46080 (3.5%) | 7 | 46240 (3.6%) | 8 | 45942 (3.5%) | 8 | 50136 (3.6%) | 7 | 50760 (3.5%) | 8 | 44484 (3.3%) | 8 | 48819 (3.2%) | 8 | 53770 (3.1%) | 8 | 59768 (2.9%) | 8 | 63472 (2.8%) | 7 |
| Otolaryngology | 484654 (3.1%) | 9 | 47031 (3.6%) | 6 | 47004 (3.7%) | 6 | 46429 (3.6%) | 7 | 47141 (3.3%) | 9 | 47735 (3.3%) | 9 | 42780 (3.2%) | 9 | 46182 (3.1%) | 9 | 49734 (2.9%) | 9 | 54272 (2.7%) | 9 | 56346 (2.5%) | 9 |
| Dental Providers | 257858 (1.6%) | 10 | 22560 (1.7%) | 12 | 22715 (1.8%) | 12 | 23091 (1.8%) | 12 | 24949 (1.8%) | 11 | 25164 (1.8%) | 10 | 23347 (1.7%) | 10 | 25956 (1.7%) | 10 | 27916 (1.6%) | 11 | 30406 (1.5%) | 11 | 31754 (1.4%) | 11 |
| **Short-term oral corticosteroids use** | | | | | | | | | | | | | | | | | | | | | | |
| **Physician specialties, *n* (%)** | | | | | | | | | | | | | | | | | | | | | | |
| Family Practice | 3210015 (25.0%) | 1 | 292926 (27.2%) | 1 | 295417 (27.9%) | 1 | 293388 (27.3%) | 1 | 313730 (27.3%) | 1 | 310360 (26.3%) | 1 | 283435 (25.4%) | 1 | 304843 (24.5%) | 1 | 341731 (23.8%) | 1 | 383115 (22.9%) | 1 | 391070 (21.5%) | 1 |
| Internal Medicine | 1448382 (11.3%) | 2 | 131121 (12.2%) | 2 | 131498 (12.4%) | 2 | 129607 (12.1%) | 2 | 138626 (12.0%) | 2 | 139712 (11.8%) | 2 | 125164 (11.2%) | 2 | 137520 (11.0%) | 3 | 155342 (10.8%) | 3 | 177018 (10.6%) | 3 | 182774 (10.0%) | 4 |
| Nurse Practitioner | 1256432 (9.8%) | 3 | 36614 (3.4%) | 7 | 45386 (4.3%) | 5 | 57414 (5.4%) | 5 | 76177 (6.6%) | 5 | 93471 (7.9%) | 3 | 108687 (9.7%) | 3 | 140707 (11.3%) | 2 | 186925 (13.0%) | 2 | 236217 (14.1%) | 2 | 274834 (15.1%) | 2 |
| Physician Assistant | 981896 (7.7%) | 4 | 34055 (3.2%) | 8 | 42258 (4.0%) | 6 | 53221 (5.0%) | 6 | 68473 (5.9%) | 6 | 81189 (6.9%) | 5 | 91499 (8.2%) | 4 | 115457 (9.3%) | 4 | 139635 (9.7%) | 4 | 168336 (10.1%) | 4 | 187773 (10.3%) | 3 |
| Emergency Medicine | 876695 (6.8%) | 5 | 73291 (6.8%) | 4 | 71201 (6.7%) | 4 | 75215 (7.0%) | 4 | 84157 (7.3%) | 3 | 85371 (7.2%) | 4 | 82584 (7.4%) | 5 | 88899 (7.1%) | 5 | 98999 (6.9%) | 5 | 109228 (6.5%) | 5 | 107750 (5.9%) | 5 |
| Pediatrics | 739557 (5.8%) | 6 | 103541 (9.6%) | 3 | 91011 (8.6%) | 3 | 86072 (8.0%) | 3 | 81859 (7.1%) | 4 | 73582 (6.2%) | 6 | 63798 (5.7%) | 6 | 60784 (4.9%) | 6 | 59613 (4.2%) | 6 | 61426 (3.7%) | 6 | 57871 (3.2%) | 6 |
| Surgery | 457297 (3.6%) | 7 | 40875 (3.8%) | 5 | 41391 (3.9%) | 7 | 41088 (3.8%) | 7 | 44761 (3.9%) | 7 | 46072 (3.9%) | 7 | 40420 (3.6%) | 7 | 44054 (3.5%) | 7 | 48525 (3.4%) | 7 | 53526 (3.2%) | 7 | 56585 (3.1%) | 7 |
| Otolaryngology | 417374 (3.3%) | 8 | 40148 (3.7%) | 6 | 40300 (3.8%) | 8 | 39959 (3.7%) | 8 | 40009 (3.5%) | 8 | 41207 (3.5%) | 8 | 37251 (3.3%) | 8 | 39910 (3.2%) | 8 | 42876 (3.0%) | 8 | 46955 (2.8%) | 8 | 48759 (2.7%) | 8 |
| Dental Providers | 245999 (1.9%) | 9 | 21484 (2.0%) | 11 | 21713 (2.0%) | 9 | 22002 (2.1%) | 9 | 23593 (2.0%) | 9 | 24043 (2.0%) | 9 | 22395 (2.0%) | 9 | 24797 (2.0%) | 9 | 26660 (1.9%) | 9 | 29044 (1.7%) | 9 | 30268 (1.7%) | 9 |
| Allergy & Immunology | 178273 (1.4%) | 10 | 21930 (2.0%) | 9 | 20331 (1.9%) | 10 | 19723 (1.8%) | 10 | 18455 (1.6%) | 10 | 18621 (1.6%) | 10 | 15748 (1.4%) | 10 | 15814 (1.3%) | 10 | 15892 (1.1%) | 12 | 16080 (1.0%) | 13 | 15679 (0.9%) | 15 |
| **Medium-term oral corticosteroids use** | | | | | | | | | | | | | | | | | | | | | | |
| **Physician specialties, *n* (%)** | | | | | | | | | | | | | | | | | | | | | | |
| Family Practice | 285382 (18.8%) | 1 | 24404 (19.2%) | 1 | 23530 (19.7%) | 1 | 25647 (19.8%) | 1 | 28512 (19.6%) | 1 | 26687 (19.2%) | 1 | 23825 (19.3%) | 1 | 27513 (19.1%) | 1 | 30888 (18.7%) | 1 | 36547 (18.4%) | 1 | 37829 (16.9%) | 1 |
| Internal Medicine | 207203 (13.7%) | 2 | 16721 (13.1%) | 2 | 16424 (13.7%) | 2 | 18275 (14.1%) | 2 | 19802 (13.6%) | 2 | 19250 (13.8%) | 2 | 16737 (13.5%) | 2 | 20377 (14.2%) | 2 | 23244 (14.1%) | 2 | 27139 (13.7%) | 2 | 29234 (13.0%) | 2 |
| Rheumatology | 102882 (6.8%) | 3 | 9617 (7.5%) | 3 | 8691 (7.3%) | 3 | 9346 (7.2%) | 3 | 9396 (6.5%) | 3 | 10175 (7.3%) | 3 | 9199 (7.4%) | 3 | 9932 (6.9%) | 4 | 11183 (6.8%) | 4 | 12304 (6.2%) | 5 | 13039 (5.8%) | 5 |
| Nurse Practitioner | 98609 (6.5%) | 4 | 3120 (2.4%) | 13 | 3431 (2.9%) | 9 | 4691 (3.6%) | 6 | 6577 (4.5%) | 4 | 7302 (5.2%) | 4 | 7817 (6.3%) | 4 | 10236 (7.1%) | 3 | 13666 (8.3%) | 3 | 18543 (9.4%) | 3 | 23226 (10.4%) | 3 |
| Physician Assistant | 74305 (4.9%) | 5 | 2598 (2.0%) | 15 | 3158 (2.6%) | 13 | 3965 (3.1%) | 10 | 5560 (3.8%) | 9 | 5996 (4.3%) | 6 | 6396 (5.2%) | 5 | 8438 (5.9%) | 5 | 10429 (6.3%) | 5 | 12835 (6.5%) | 4 | 14930 (6.7%) | 4 |
| Pulmonology | 69002 (4.6%) | 6 | 5961 (4.7%) | 4 | 5428 (4.5%) | 5 | 5572 (4.3%) | 4 | 6247 (4.3%) | 6 | 6379 (4.6%) | 5 | 5789 (4.7%) | 6 | 6602 (4.6%) | 6 | 7685 (4.7%) | 6 | 9132 (4.6%) | 6 | 10207 (4.6%) | 6 |
| Otolaryngology | 57088 (3.8%) | 7 | 5747 (4.5%) | 5 | 5589 (4.7%) | 4 | 5470 (4.2%) | 5 | 6047 (4.2%) | 7 | 5601 (4.0%) | 7 | 4728 (3.8%) | 7 | 5286 (3.7%) | 8 | 5881 (3.6%) | 8 | 6282 (3.2%) | 8 | 6457 (2.9%) | 9 |
| Emergency Medicine | 55352 (3.7%) | 8 | 4111 (3.2%) | 8 | 3891 (3.3%) | 7 | 4522 (3.5%) | 8 | 5837 (4.0%) | 8 | 5424 (3.9%) | 8 | 4600 (3.7%) | 8 | 5512 (3.8%) | 7 | 6241 (3.8%) | 7 | 7568 (3.8%) | 7 | 7646 (3.4%) | 7 |
| Allergy & Immunology | 40678 (2.7%) | 9 | 5151 (4.0%) | 6 | 4658 (3.9%) | 6 | 4547 (3.5%) | 7 | 4697 (3.2%) | 10 | 4025 (2.9%) | 9 | 3340 (2.7%) | 9 | 3429 (2.4%) | 11 | 3598 (2.2%) | 12 | 3715 (1.9%) | 12 | 3518 (1.6%) | 13 |
| Surgery | 37363 (2.5%) | 10 | 3233 (2.5%) | 12 | 3262 (2.7%) | 11 | 3364 (2.6%) | 14 | 3912 (2.7%) | 11 | 3385 (2.4%) | 14 | 3058 (2.5%) | 10 | 3559 (2.5%) | 9 | 3918 (2.4%) | 10 | 4666 (2.4%) | 10 | 5006 (2.2%) | 10 |
| **Long-term oral corticosteroids use** | | | | | | | | | | | | | | | | | | | | | | |
| **Physician specialties, *n* (%)** | | | | | | | | | | | | | | | | | | | | | | |
| Rheumatology | 344334 (26.6%) | 1 | 34245 (28.8%) | 1 | 29347 (29.1%) | 1 | 29337 (28.6%) | 1 | 32803 (28.7%) | 1 | 32768 (28.6%) | 1 | 30061 (28.5%) | 1 | 33479 (27.6%) | 1 | 37302 (26.3%) | 1 | 40538 (24.0%) | 1 | 44454 (21.6%) | 1 |
| Internal Medicine | 163919 (12.7%) | 2 | 13493 (11.3%) | 2 | 11883 (11.8%) | 2 | 12996 (12.7%) | 2 | 14612 (12.8%) | 2 | 14372 (12.6%) | 2 | 13656 (12.9%) | 2 | 15765 (13.0%) | 2 | 18745 (13.2%) | 2 | 22616 (13.4%) | 2 | 25781 (12.6%) | 2 |
| Family Practice | 150830 (11.7%) | 3 | 12773 (10.7%) | 3 | 11016 (10.9%) | 3 | 11492 (11.2%) | 3 | 12938 (11.3%) | 3 | 12964 (11.3%) | 3 | 12768 (12.1%) | 3 | 14697 (12.1%) | 3 | 17446 (12.3%) | 3 | 20946 (12.4%) | 3 | 23790 (11.6%) | 3 |
| Pulmonology | 63271 (4.9%) | 4 | 5148 (4.3%) | 5 | 4682 (4.6%) | 4 | 4702 (4.6%) | 4 | 5313 (4.7%) | 4 | 5484 (4.8%) | 4 | 4688 (4.4%) | 4 | 6002 (5.0%) | 4 | 7401 (5.2%) | 5 | 9017 (5.3%) | 5 | 10834 (5.3%) | 5 |
| Nurse Practitioner | 56026 (4.3%) | 5 | 1846 (1.6%) | 12 | 1906 (1.9%) | 10 | 2483 (2.4%) | 9 | 3387 (3.0%) | 8 | 3845 (3.4%) | 5 | 4252 (4.0%) | 5 | 5772 (4.8%) | 5 | 7890 (5.6%) | 4 | 10149 (6.0%) | 4 | 14496 (7.1%) | 4 |
| Nephrology | 40236 (3.1%) | 6 | 5374 (4.5%) | 4 | 4011 (4.0%) | 6 | 3697 (3.6%) | 6 | 4018 (3.5%) | 5 | 3811 (3.3%) | 6 | 3315 (3.1%) | 6 | 3464 (2.9%) | 7 | 3833 (2.7%) | 8 | 4176 (2.5%) | 8 | 4537 (2.2%) | 8 |
| Specialist | 37874 (2.9%) | 7 | 4440 (3.7%) | 7 | 4019 (4.0%) | 5 | 3862 (3.8%) | 5 | 3966 (3.5%) | 6 | 3642 (3.2%) | 7 | 3220 (3.1%) | 7 | 3447 (2.8%) | 8 | 3783 (2.7%) | 9 | 3632 (2.1%) | 10 | 3863 (1.9%) | 10 |
| Gastroenterology | 36888 (2.9%) | 8 | 4576 (3.8%) | 6 | 3883 (3.8%) | 7 | 3538 (3.4%) | 7 | 3915 (3.4%) | 7 | 3553 (3.1%) | 8 | 3108 (2.9%) | 9 | 3135 (2.6%) | 10 | 3455 (2.4%) | 10 | 3679 (2.2%) | 9 | 4046 (2.0%) | 9 |
| Physician Assistant | 36510 (2.8%) | 9 | 1492 (1.3%) | 16 | 1584 (1.6%) | 15 | 1953 (1.9%) | 10 | 2494 (2.2%) | 10 | 2949 (2.6%) | 10 | 3219 (3.0%) | 8 | 3914 (3.2%) | 6 | 4855 (3.4%) | 6 | 6020 (3.6%) | 6 | 8030 (3.9%) | 7 |
| Hematology & Oncology | 34521 (2.7%) | 10 | 1761 (1.5%) | 15 | 1612 (1.6%) | 13 | 1836 (1.8%) | 12 | 2159 (1.9%) | 11 | 2494 (2.2%) | 11 | 2435 (2.3%) | 11 | 3163 (2.6%) | 9 | 4232 (3.0%) | 7 | 5612 (3.3%) | 7 | 9217 (4.5%) | 6 |
